# Control of *Vibrio parahaemolyticus* in Seafood Using the Combination of Lytic Phages and Citric Acid

**DOI:** 10.3390/foods14010037

**Published:** 2024-12-26

**Authors:** Xiaoshuang Zheng, Lu Gao, Lei Yuan, Caowei Chen, Zhenquan Yang

**Affiliations:** 1 School of Biological and Chemical Engineering, Yangzhou Polytechnic College, Yangzhou 225009, China; 102012@yzpc.edu.cn; 2 Yangzhou Engineering Research Center of Agricultural Products Intelligent Measurement and Control & Cleaner Production, Yangzhou Polytechnic College, Yangzhou 225009, China; 3 College of Food Science and Engineering, Yangzhou University, Yangzhou 225127, China; gaolu@yzu.edu.cn (L.G.); leiyuan@yzu.edu.cn (L.Y.); 17761806270@163.com (C.C.)

**Keywords:** *Vibrio parahaemolyticus*, phage, citric acid, combined treatment, food safety

## Abstract

*Vibrio parahaemolyticus* is a key foodborne pathogen in seafood that poses health risks to consumers. The application of phages and organic acids is considered an alternative strategy for controlling bacterial contamination in foods. In the present study, the genome features of five previously isolated virulent *V. parahaemolyticus* phages (VPpYZU64, VPpYZU68, VPpYZU81, VPpYZU92, and VPpYZU110) were characterized, and their bacteriostatic effects in combination with citric acid were analyzed. Genome sequencing of the five phages showed a total genome length of 76,153–144,768 bp. No virulent or drug-resistant genes were detected in the five phages. Bacterial inhibition testing of salmon fillets stored at 25 °C for 12 h showed that the number of *V. parahaemolyticus* decreased by 2.02 and 3.84 log CFU/g after treatment with a phage mixture, *VPp*MIx, and the combination of phage mixture *VPp*MIx and citric acid. In addition, phage VPpYZU64 combined with 600 μg/mL citric acid exhibited the highest biofilm reduction rate for *V. parahaemolyticus*. Collectively, our results show that combining phages and citric acid is a natural and efficient method of controlling *V. parahaemolyticus* growth in seafood.

## 1. Introduction

*Vibrio parahaemolyticus* is the main pathogen responsible for the transmission of bacterial gastroenteritis from seafood [1]. They can grow at a temperature range of 8–45 °C, with an optimal growth temperature of 37–39 °C [2]. *V. parahaemolyticus* readily forms biofilms on seafood and surfaces in contact with serving utensils, increasing the risk of *V. parahaemolyticus* infection in humans as well as cross-contamination with other food products [3]. Currently, *V. parahaemolyticus* reproduction is mainly controlled using low temperatures during seafood storage and transportation. However, temperature fluctuations in the cold chain or exposure to the appropriate temperature range for *V. parahaemolyticus* growth can cause rapid proliferation of *V. parahaemolyticus* to pathogenic levels. Therefore, using bacteriostats to reduce the risk of *V. parahaemolyticus* proliferation is a safe seafood storage method throughout the supply chain. Antibiotics have been conventionally used to control *V. parahaemolyticus* growth; however, they lead to the emergence of drug-resistant bacteria and the accumulation of antibiotic residues in the environment [4].

Exploring and developing alternatives to antibiotics for the control of *V. parahaemolyticus* is particularly important. Among these alternatives, organic acids and phages have attracted attention because of their unique antimicrobial mechanisms. Organic acids are widely used as antimicrobial agents within the food industry. Their use in specific foods is associated with their chemical formula, pKa, minimum inhibitory concentration, molecular weight, the physiological characteristics of target microorganisms, acid treatment duration, and the type of food [5]. Organic acids are generally recognized as safe (GRAS) by the US Food and Drug Administration. Foodborne pathogens, including *Staphylococcus aureus*, *Bacillus* spp., *Listeria monocytogenes*, *Salmonella*, *Escherichia coli*, and *Pseudomonas aeruginosa*, are inhibited by organic acids [6,7]. Guo, He, Wang, and Yang [8] showed that 1% citric acid, 1.5% acetic acid, and 1.5% lactic acid reduced the number of *Salmonella* spp. inoculated on cucumber slices by 1.7 to 2.5 log CFU/g. The main organic acid bacteriostats used in seafood are ascorbic acid, citric acid, acetic acid, lactic acid, and sodium or potassium salts. Organic acid bacteriostats are usually prepared as aqueous solutions at concentrations of 0.05–2.5%, where seafood is immersed for approximately 10–15 min. Following immersion in these solutions, seafood is stored in alternate layers of ice [9]. Citric acid is a naturally occurring, water-soluble, ternary carboxylic acid approved for addition to various foods. The Food and Agriculture Organization does not specify a restriction on the acceptable daily intake of citric acid, which has been approved for use in fresh meat and processed poultry. Citric acid has been shown to lower the pH of the cell cytoplasm [10]. Bacteria consume large amounts of ATP to maintain homeostasis, which leads to energy depletion and eventual cell death. García-Soto, Böhme, Barros-Velázquez, and Aubourg [11] applied an aqueous solution of citric acid (1.25 g/L) and lactic acid (0.5 g/L) in the chilled storage of megrim for 13 days, and the application exhibited good bacterial growth inhibition and good sensory acceptance.

Another alternative method is the application of phages. Phages are widely distributed, simple to prepare, inexpensive, and cause no harm to the environment. In recent years, phages have played an important role as biological control agents in food preservation and safety management [12,13]. However, phages are species- or strain-specific and have a limited spectrum of bacterial growth inhibition activity. Furthermore, bacteria may develop resistance during phage lysis in the host, resulting in a short period of effective bacterial inhibition and phage resistance, in addition to environmental and regulatory issues that remain unresolved [14,15,16]. The pH, temperature, and chemical composition of food affect phage efficacy [17]. Currently, screening for efficient broad-spectrum phages and preparing phage cocktails are the primary strategies for widening the spectrum of bacterial inhibition and minimizing the development of resistance [18].

The combination of phage-based biological controls and conventional antimicrobial agents is also actively explored. Oechslin et al. [19] found that 10^8^ plaque-forming units (PFU)/mL of phages were highly synergistic with ciprofloxacin against *P. aeruginosa*. A single dose of phages killed 2.50 log CFU/g of bacteria in rats within 6 h, which was comparable to the effects of ciprofloxacin. The combined use of phages and ciprofloxacin killed more than 6 log CFU/g of bacteria. Moye et al. [20] treated *Salmonella*-contaminated fresh produce with phages combined with chlorine or peracetic acid. They found that the combination of peracetic acid and phage showed increased bactericidal efficacy compared to each treatment alone while having minimal effects on the microbiomes of produce. Wang et al. [21] used phages, nisin, potassium sorbate, and combinations of these agents to reduce the growth of *Salmonella* and spoilage bacteria in chilled fresh pork. Their results showed that combinations of these three bacteriostats significantly lowered the total viable basic nitrogen, total volatile counts, and levels of thiobarbituric acid-reactive substances. Due to the diversity of bacteria and phage control time limit, a single phage is not enough to control infection, thus requiring the combination of a phage cocktail and a low dose of citric acid for greater antibacterial effects. Pelyuntha et al. [22] combined a mixture of two *Salmonella* phage strains with 0.25% propionic acid and applied it to a chicken model contaminated with *Salmonella*. After 72 h of storage at 4 °C, a significant decrease of 1.4 log CFU/g of *Salmonella* in chicken was observed. This experiment confirmed the effectiveness of combining phages and organic acids in a chicken model. Eraclio et al. [23] combined *Listeria monocytogenes* with buffered vinegar on ready-to-eat turkey slices contaminated with four *Listeria* strains, storing them under vacuum conditions at 4 °C. They found that the growth of *L. monocytogenes* was inhibited below the detection limit within 120 days. When *L. monocytogenes* was added to potassium lactate (72.8%)/sodium diacetate (5.2%)-treated turkey slices, no *L. monocytogenes* was detected within 30 days. Hence, their experimental results confirmed that phage combination can effectively inhibit *L. monocytogenes* during the processing of ready-to-eat foods.

Moreover, bacteriostatic agents alone may not be sufficient to eliminate contaminating microbes present in food or on food surfaces. The combined application of phages and organic acids can produce a synergistic effect, thereby significantly reducing bacterial populations in a short period and enhancing the control of pathogens. Organic acids, their salts, and phages are GRAS [22,24]; therefore, their combinations can be used as a viable food safety intervention. Phages and organic acids are relatively cheap. Moreover, they are biodegradable substances with low environmental impact and do not cause long-term harm to the ecosystem. Overall, the combined use of phages and organic acids in food matrices demonstrated significant potential and advantages, which not only enhanced the control of foodborne pathogens but also combined safety, economy, and environmental friendliness.

Herein, this study aimed to develop an alternative strategy to control the contamination of *V. parahaemolyticus* in foods by combining citric acid and *V. parahaemolyticus* phages. The genomic features of five previously isolated virulent *V. parahaemolyticus* phages (VPpYZU64, VPpYZU68, VPpYZU81, VPpYZU92, and VPpYZU110) were characterized, and their bacteriostatic effects in combination with citric acid in culture media as well as in a salmon model were analyzed. This study provides evidence for the efficacy of a novel bacteriostatic agent in reducing the risk of *V. parahaemolyticus* contamination in seafood.

## 2. Materials and Methods

### 2.1. Bacterial Strains and Culture Conditions

In our previous study, we isolated five *V. parahaemolyticus* phages that maintained high lytic activity at 60 °C at pH levels ranging from 4.0 to 10.0 [25]. Five *V. parahaemolyticus* strains, VpYZU64, VpYZU68, VpYZU81, VpYZU92, and VpYZU110, and their corresponding phages, VPpYZU64, VPpYZU68, VPpYZU81, VPpYZU92, and VPpYZU110, respectively, were isolated from local food markets in Yangzhou and stored at the Food Quality and Safety Laboratory of Yangzhou University at −70 °C. An inoculation loop was dipped into the defrosted bacterial solution, streaked onto thiosulfate–citrate–bile salt–sucrose (TCBS, Binhe Microorganism Reagent Co., Ltd., Hangzhou, China) agar medium, and incubated at 37 °C for 18 h. Single colonies were picked, inoculated into 5 mL of 3% Luria-Bertani (LB, Sigma-Aldrich, Poole, UK) broth supplemented with 3% (*w*/*v*) NaCl to create LBS, and incubated at 37 °C for 6 h with shaking at 150 rpm until further use.

### 2.2. Preparation of Phages

Phages were prepared as previously described [26]. Briefly, 1 mL of the host bacterial suspension (10^8^ CFU/mL) and 500 μL of the stored phage suspension were mixed and allowed to stand for 10 min at 37 °C. The 1.5 mL mixture was inoculated into 30 mL of LBS broth and incubated for 6 h at 37 °C with shaking at 150 rpm. The culture was then centrifuged for 10 min at 10,000× *g* (Eppendorf 5810R; Hamburg, Germany) at 4 °C. The supernatant was filtered through a 0.22-μm filter (Millex, Merck Millipore Ltd., Co., Cork, Ireland), and the phage solution was stored at 4 °C until use. Phage activity was determined using a spot assay [27], phage titer was determined using the double-layer agar method as previously described [28], and plaque formation was documented by capturing images from three independent plates.

A *VPp*MIx mixed phage suspension was prepared as previously described [29]. Briefly, the five phage suspensions were mixed at a ratio of 1:1:1:1:1 with a phage titer of 10^6^ PFU/mL for each phage and stored until further use.

### 2.3. Genomic Analysis of the Phages

The extraction and purification of genomic DNA from the five phage strains were performed as previously described by Yang et al. [30]. Briefly, phage genomic DNA was extracted and purified using a phage DNA purification kit (AB1141, Beijing ABigen Biotechnology Co., Ltd., Beijing, China). The extracted phage DNA was subjected to agarose gel electrophoresis, and its concentration was quantitatively determined using a Qubit^®^ dsDNA HS Analysis Kit and a Qubit 2.0 fluorometer (Life Technologies, Carlsbad, CA, USA).

An Illumina HiSeq 4000 sequencing system was used for paired-end sequencing of the phage sample DNA. To improve the accuracy of genome assembly, low-quality regions from the raw Illumina sequencing data were trimmed. The optimized sequences were assembled with multiple Kmer parameters using ABySS assembler software 2.0 (http://www.bcgsc.ca/platform/bioinfo/software/abyss, accessed on 5 February 2018) to obtain the optimal assembly results.

The protein sequences of the predicted genes were aligned with the Kyoto Encyclopedia of Genes and Genomes, Swiss-Prot, Clusters of Orthologous Groups of Proteins, and Gene Ontology databases using BlastP (alignment criteria: E ≤ 1 × 10^−5^). Annotation data of the predicted genes were obtained.

A BLAST homology comparison of the whole-genome sequences of *Vibrio* phages was performed on the NCBI, and 57 phage strains with high homology to *Vibrio* phages were obtained. The phage linear dsDNA genome was schematically mapped using IBS 1.0.2 software (http://ibs.biocuckoo.org/index.php, accessed on 26 October 2024).

### 2.4. Growth Curve of V. parahaemolyticus in Citric Acid-LBS Medium

Citric acid-LBS medium was prepared by accurately weighing citric acid and adding it to LBS medium at final concentrations of 200, 400, 600, and 800 μg/mL. Briefly, 200 μL of normal saline-diluted suspensions of *V. parahaemolyticus* strains VpYZU64, VpYZU68, VpYZU81, VpYZU92, and VpYZU110 was inoculated into 9.8 mL of citric acid-LBS culture medium (10^5^ CFU/mL) and mixed. Next, 200 μL of this bacterial suspension was added to a sterile honeycomb plate, placed in an automated microbiology growth curve analysis system (Bioscreen, Turku, Finland), and cultured at 37 °C for 24 h. Five biological replicates were performed for each group. The optical density (OD) of cultures at 600 nm was measured every 2 h, and the average OD was used to construct growth curves for *V. parahaemolyticus* strains under different citric acid concentrations, reflecting the inhibitory effect of citric acid on *V. parahaemolyticus* growth.

### 2.5. Inhibition of V. parahaemolyticus Growth with the Combination of Phage and Citric Acid

Strains of VpYZU64, VpYZU68, VpYZU81, VpYZU92, and VpYZU110 in the logarithmic phase were collected and diluted to 10^3^ CFU/mL with saline. Equal volumes of these suspensions were mixed to form a mixed strain suspension. Next, 200 μL of this mixed strain suspension was inoculated into 9.8 mL of LBS broth and mixed thoroughly. The experiment was divided into four groups: an untreated control group, a phage treatment group (in which 200 μL of *VPp*MIx was added to the inoculation tube), a citric acid treatment group (in which 200 μL of citric acid solution was added to the inoculation tube to a final concentration of 500 μg/mL), and a phage + citric acid treatment group (in which 200 μL of 500 μg/mL citric acid solution and 200 μL of *VPp*MIx were added to the inoculation tube). After thorough mixing, the samples were placed in a constant-temperature incubator at 25 °C or 37 °C. Samples were taken at 0, 6, 12, 18, 24, 30, and 36 h at 25 °C or 0, 3, 6, 9, at 12 h at 37 °C. The TCBS plate dilution method [31] was used to determine the number of *V. parahaemolyticus*. Phage titer was determined using the double-layer agar method. Each group was established in biological triplicates, and the average was used to compare the inhibitory effects in each treatment group.

### 2.6. Inhibition of V. parahaemolyticus in Salmon by the Combination Treatment of Phage and Citric Acid

Fresh salmon meat was collected, aseptically cut into slices with sizes of 5 × 5 × 1 cm, weighed for 25 g, and sterilized using UV irradiation for 30 min (15 min on each side). Salmon fillets were contaminated using the surface inoculation method, as described previously [31]. The inoculation volume of *Vp*MIx, *VPp*MIx, citric acid solution, and normal saline was 200 μL. The inoculum was spread uniformly on the surface of the fillets by spotting 10 μL of each solution at 20 random points.

After the salmon fillets were inoculated with *Vp*MIx, they were divided evenly into four groups: control group (inoculated with saline twice), phage treatment group (inoculated with *VPp*MIx), citric acid treatment group (inoculated with citric acid solution), and phage + citric acid treatment group (inoculated sequentially with *VPp*MIx followed by a citric acid solution). The phage control group comprised uncontaminated fillets inoculated with *VPp*MIx. Low-temperature air drying was performed for 15 min after each inoculation to allow binding of the inoculum. Fillets from each treatment group were placed in sterile plates and incubated at 25 °C or 37 °C. To investigate the risk prevention and control of *V. parahaemolyticus* growth in salmon exposed to conventional room temperature (25 °C) and at an extreme temperature (37 °C) where *V. parahaemolyticus* grows optimally.

The salmon pieces were collected at 0, 6, 12, 18, 24, 30, and 36 h at 25 °C or 0, 3, 6, 9, and 12 h at 37 °C. Fish samples were homogenized (Stomacher 80 paddle blender, Seward, UK) in 20 mL saline, and the resulting homogenate was diluted through a saline gradient, spread onto TCBS plates, and incubated at 37 °C for 18 h. Each total cell number was counted and expressed as log CFU/g. The phage titer (log PFU/g) was determined using the double-layer agar method. Each group was established in biological triplicates, and the average was used to compare the inhibitory effects in each treatment group.

### 2.7. Inhibition of Biofilm Formation Using the Phage and Citric Acid Combination Treatment

Phage strain VPpYZU64 (10^9^ PFU/mL), which had the highest lytic capacity, was combined with 600 µg/mL citric acid to observe the elimination of VpYZU64 (10^7^ CFU/mL) biofilms. The bacterial biofilm was quantified using the crystal violet method, as previously reported [32]. Briefly, 200 μL (10^7^ CFU/mL) of a bacterial suspension diluted with culture medium was added to each well of a 96-well plate and incubated at 37 °C for 24 h. The culture solution was discarded, and each well was washed three times with sterilized phosphate-buffered saline (PBS) (10 mM Na_2_HPO_4_/NaH_2_PO_4_, 150 mM NaCl, pH 7.4). Next, 200 μL of phage solution (10^9^ PFU/mL), 200 μL of 600 μg/mL of citric acid, or 200 μL of phage solution and 600 μg/mL of citric acid (*v*/*v*, 1:1) was added to the wells of the experimental groups, while 200 μL of LBS broth was added to the wells of the control group, and the plates were incubated at 37 °C for 6 h. After incubation, each well was washed three times with sterile PBS and air-dried in a biosafety cabinet. Next, 200 μL of methanol solution (Sangon Biotech Co., Ltd., Shanghai, China) was added to each well and incubated for 15 min to fix the cells, followed by staining with 0.20% crystal violet for 30 min. The wells were washed with PBS, and 200 μL of 33% acetic acid solution was added to each well to dissolve the biofilms formed, after which the absorbance of the solution at 600 nm was measured using a spectrophotometer UV-7504C (Xinmao Instrument Co., Ltd., Shanghai, China). Five biological replicates were performed for each experiment.

### 2.8. Field Emission Scanning Electron Microscopy of V. parahaemolyticus Biofilms

Field emission scanning electron microscopy (FESEM) was performed to observe the biofilm structures, as described by Baños et al. [33]. Briefly, 1 mL of the VpYZU64 bacterial suspension (10^7^ CFU/mL) was added to each well of a 24-well plate, a sterile coverslip was placed in each well, and the plate was incubated at 37 °C for 24 h. After incubation, the bacterial suspension was discarded, and loose bacteria were removed by washing with PBS. Then, 1 mL of phage suspension (10^9^ PFU/mL), 600 µg/mL citric acid solution, or 1:1 phage suspension and 600 µg/mL citric acid solution was added to the experimental groups, whereas 1 mL LBS broth was added to the control group, and the plates were incubated at 37 °C for another 6 h. The coverslips were then removed, washed three times with PBS, fixed with 2.50% glutaraldehyde solution overnight at 4 °C, washed again with PBS, and dehydrated through an ethanol gradient (30, 50, 60, 70, 80, 90, and 100%) for 15 min at each step. Next, the coverslips were immersed in ethanol containing anhydrous sodium sulfate, dried, immobilized, sputter-coated with gold, and imaged using FESEM (S-4800f, Hitachi, Japan).

### 2.9. Statistical Analysis

All experiments were repeated three times, and data were expressed as the mean (SD). Measurement data were analyzed using IBM SPSS Statistics 20 (IBM Corp., Armonk, NY, USA), and graphs were plotted using Origin 8.5 (OriginLab Corp., Northampton, MA, USA). Significance was analyzed using analysis of variance, with *p* < 0.05 indicating statistical significance.

## 3. Results and Discussion

### 3.1. Characterization of V. parahaemolyticus Phage Genomes

#### 3.1.1. Gene Composition

The full length, GC content, total coding gene length, and average length of the five phage genomes are shown in Table 1. The size of the coding genome ranged from 18,868 to 74,577 bp, and the GC content ranged from 22.51 to 49.28%, reflecting the biodiversity of *V. parahaemolyticus* phages. The number of coding genes ranged from 27 to 114. Genomic analysis revealed that none of the five phage strains had known virulence genes. The sequenced and assembled genome sequences of the five phage strains (VPpYZU64, VPpYZU68, VPpYZU81, VPpYZU92, and VPpYZU110) were combined with the coding gene prediction results, and circular maps of the genomes of the five phage strains were constructed (Supplementary Figure S1). The statistical results of the second-generation sequencing data for the five phage strains are presented in Supplementary Table S1. The statistical graphs of the correlation analysis between the GC content and the sequencing depth (Depth) for the five phage strains are shown in Supplementary Figure S2.

#### 3.1.2. Functional Annotation of Genes

**Figure 1:**
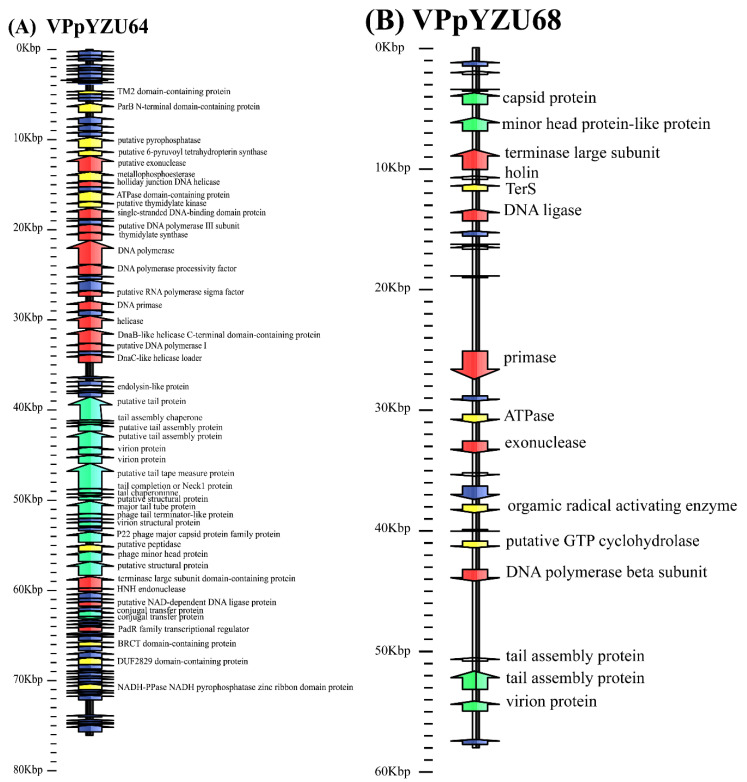

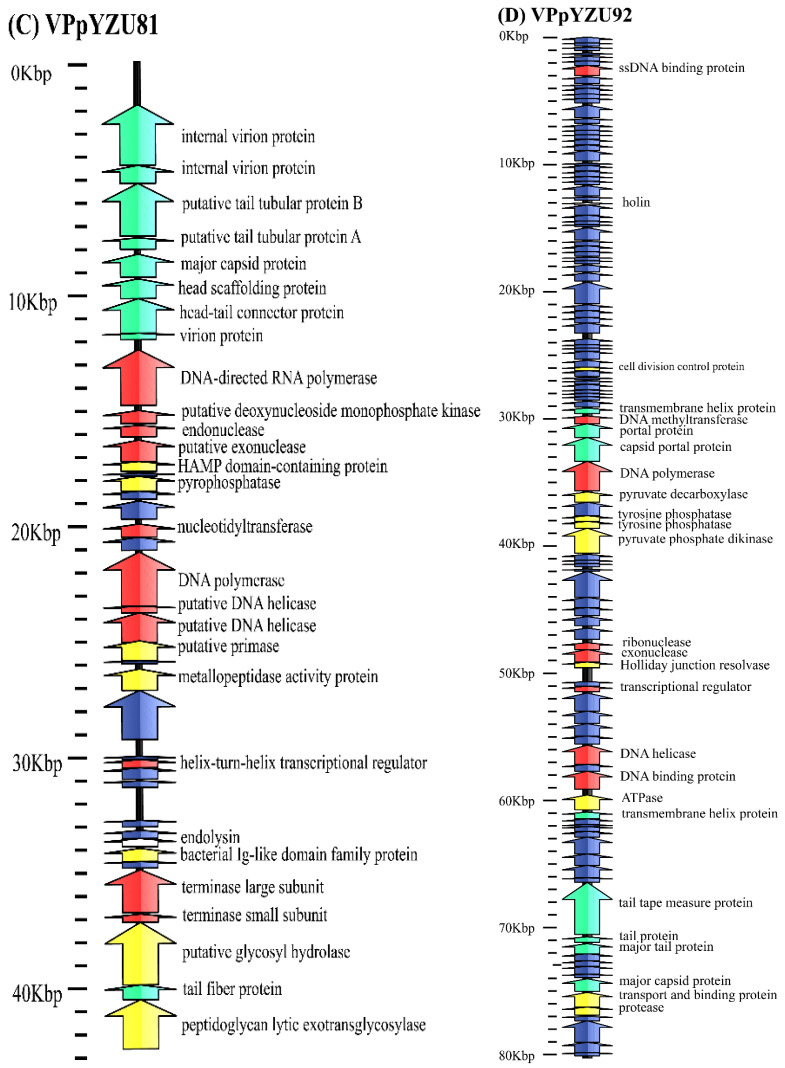

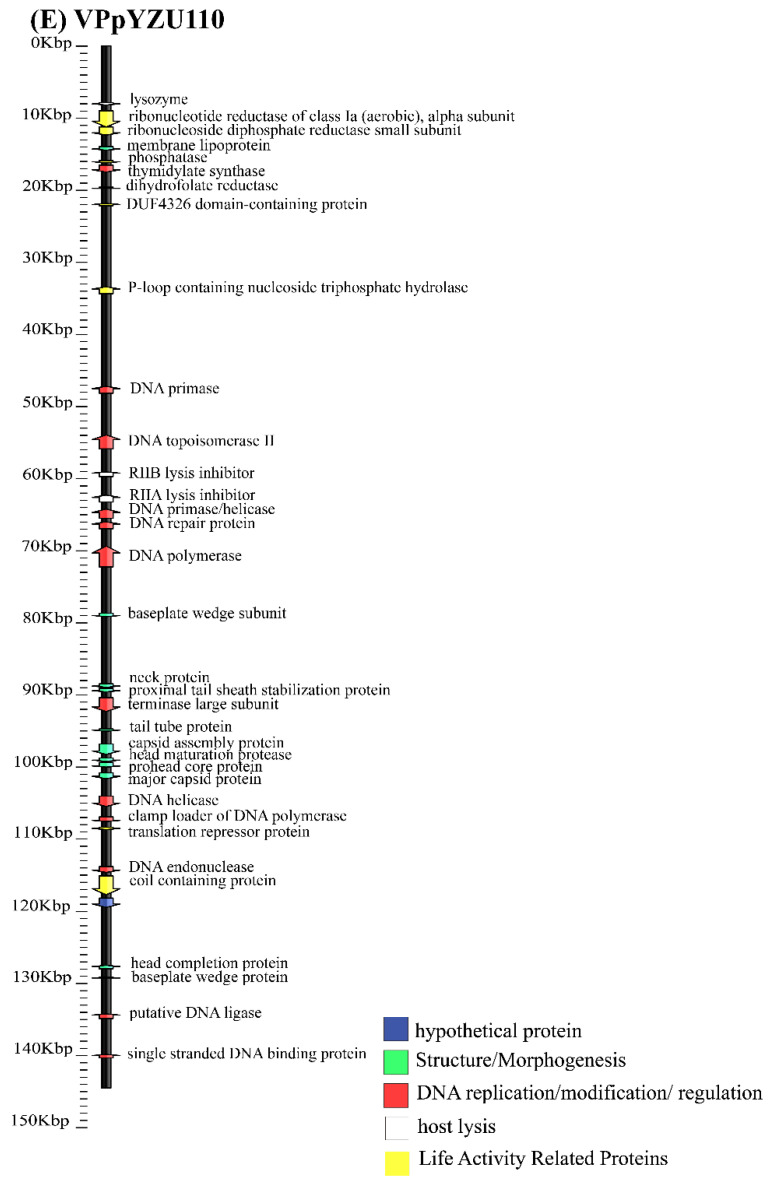
Schematic representation of the linear dsDNA genome of the phages investigated in this study: (**A**) VPpYZU64, (**B**) VPpYZU68, (**C**) VPpYZU81, (**D**) VPpYZU92, and (**E**) VPpYZU110. The positions, orientation, and function of the predicted ORFs are shown. Each ORF is represented by an arrow, and its predicted functions are indicated.

The protein sequences of the predicted genes were compared to the GeneBank database via BlastP, and a phage protein annotation map was obtained, as shown in Figure 1. The general features of the putative ORFs of the five phages are detailed in Supplementary Tables S2–S6. The functional genes and proteins they encode in the five phage annotated strains were categorized into five classes: hypothetical functional proteins, phage structural protein genes, genes related to DNA replication, genes related to life activities, and cleavage genes. The number of classified functional genes annotated for each phage strain is shown in Table 2.

Regarding the phage structural protein genes, all five phage strains had head proteins, tail proteins, and different tail proteins that exhibited different lysing abilities. Specifically, virion proteins were identified in VPpYZU64 (ORF26), VPpYZU68 (ORF26), and VPpYZU81 (ORF1, ORF2, ORF8); capsid proteins were identified in VPpYZU68 (ORF4), VPpYZU92 (ORF63, ORF108), and VPpYZU110 (ORF22, ORF25); head–tail connector protein was identified in VPpYZU81 (ORF7); portal protein was identified in VPpYZU92 (ORF62); transmembrane helix protein was identified in VPpYZU92 (ORF60, ORF92); and baseplate wedge protein was identified in VPpYZU110 (ORF17, ORF33).

All five phage strains harbored DNA replication-related genes, including those encoding DNA polymerase and endonuclease. VPpYZU64 had more DNA-assembly related genes, which may be correlated with its strong lysis ability. All five phage strains harbored lysis genes, including endolysin-like protein in VPpYZU64 (ORF45), holin in VPpYZU68 (ORF7) and VPpYZU92 (ORF28), endolysin in VPpYZU81 (ORF33), and three lysis genes in VPpYZU81, including ORF1 (lysozyme), ORF12 (RIIB lysis inhibitor), and ORF13 (RIIA lysis inhibitor). The phage strains showed variability in genes related to biological activities and did not contain known virulence or resistance-related genes, suggesting that they are safe for use as biocontrol agents.

#### 3.1.3. Phylogenetic Analysis of *V. parahaemolyticus* Phages

**Figure 2:**
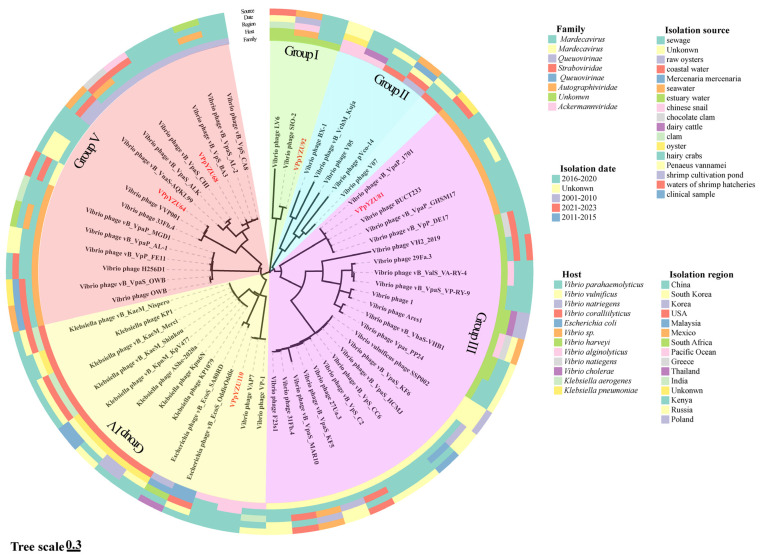
Phylogenetic tree of the *Vibrio parahaemolyticus* phages.

MegaBLAST analysis was performed to determine sequence homology between the five *V. parahaemolyticus* phage strains. The genome sequences of the 10 phages with the highest homology to each phage strain were selected, and a phylogenetic tree was constructed (Figure 2). Fifty-seven phage genome sequences with high homology were obtained.

The 62 phage strains were isolated from different sources, including sewage samples, raw oysters, seawater, estuary water, Chinese snails, dairy cattle, clinical samples, and shrimp cultivation ponds from 2001 to 2023 globally. Phages isolated from the same region were dispersed across different groups, indicating that phage distribution in nature was diverse and not restricted geographically.

The constructed phylogenetic tree is shown in Figure 1. The five phages sequenced in this study were distributed among the four groups with long branch distances. These data indicate large genetic distances among *V. parahaemolyticus* phages and suggest that the genomes of phages with the same host exhibit a high degree of genetic diversity. In the phylogenetic tree, the phage genome sequences were categorized into five groups (I–V); groups III, IV, and V were the dominant groups with 24, 13, and 16 members, respectively. In Group IV, the most diverse group, phages with different hosts were clustered together, including eight *Klebsiella* phages, two *Escherichia* phages, and three *Vibrio* phages.

Phages VPpYZU64 and VPpYZU68 from the present study were classified as Group V. The phage with the highest sequence homology and coverage with VPpYZU64 was the *Vibrio* phage VVP001 (GenBank accession no.: MG602476.1). VVP001 was isolated from the coast of Yeompo, South Korea, and exhibits host tropism for *V. vulnificus*. It had 97.79% homology and 96% coverage with VPpYZU64. The phage with the highest sequence homology and coverage with VPpYZU68 was *Vibrio* phage vB_VpaS_AL-2 (GenBank accession no.: OK349507.1). vB_VpaS_AL-2 was isolated from Mexican estuarine water and showed 97.79% homology and 95% coverage with VPpYZU68. Phage VPpYZU81 was classified as Group III. The phage sharing the highest sequence homology and coverage with VPpYZU81 is *Vibrio* phage vB_VpaP_MGD1 (GenBank accession no.: MT501516.1). vB_VpaP_MGD1 was isolated from clams in China and showed 95.63% homology and 99% coverage with VPpYZU81. Phage VPpYZU92 was classified as Group IV. The phage sharing the highest sequence homology and coverage with phage VPpYZU92 is *Vibrio* phage SIO-2 (GenBank accession number NC_016567.1). SIO-2 was isolated from Pacific coastal surface waters and showed 96.95% homology and 100% coverage with VPpYZU92. Phage VPpYZU110 was classified as Group I. The phage with the highest sequence homology and coverage with VPpYZU110 is Vibrio phage VAP7 (GenBank accession no.: NC_048765.1). VAP7 was isolated from wastewater in Beijing, China, showing 99.37% homology and 100% coverage with VPpYZU110.

### 3.2. Growth Curve of V. parahaemolyticus in Citric Acid-LBS Medium

**Figure 3:**
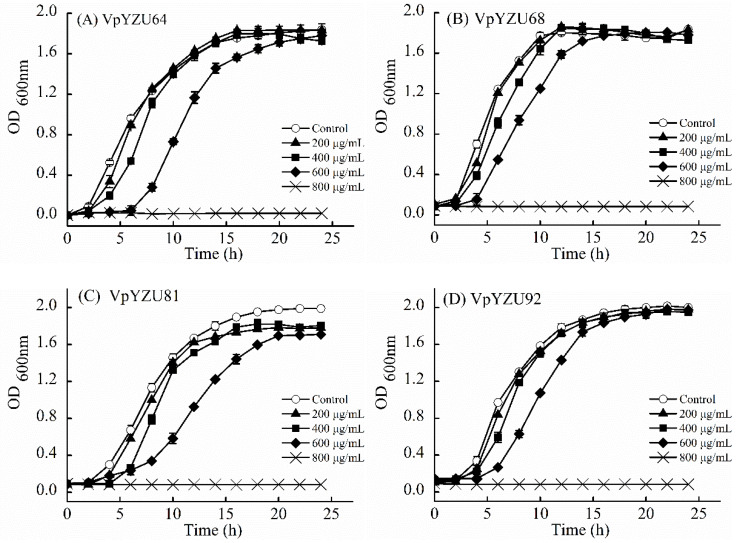

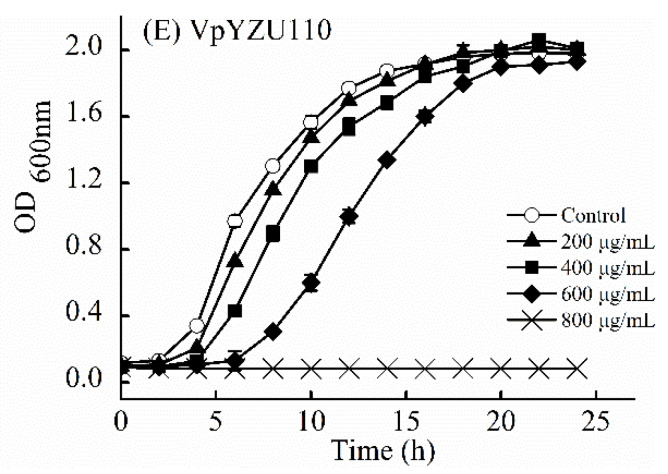
Growth curve of *Vibrio parahaemolyticus* in an acidified medium. (**A**) VpYZU64, (**B**) VpYZU68, (**C**) VpYZU81, (**D**) VpYZU92, and (**E**) VpYZU110.

The growth curves of the five *V. parahaemolyticus* host bacterial strains in the citric acid-LBS medium concentration gradient suggest a decrease in *V. parahaemolyticus* host bacterial growth with increasing citric acid concentration (Figure 3). This phenomenon is of significant biological importance for several reasons. Firstly, the antimicrobial properties of citric acid are likely responsible for the observed growth inhibition of *V. parahaemolyticus*. Secondly, the capacity of this bacterium to adapt to environmental alterations is vital for its survival in acidic environments, such as those found in food matrices, particularly in seafood preservation, where citric acid is commonly employed as a preservative [34]. The disparate responses of the various strains may be indicative of adaptive variations in acid resistance, which could be associated with their genetic diversity. Thirdly, this has implications for food safety. An understanding of the manner in which citric acid inhibits the growth of *V. parahaemolyticus* could inform strategies to optimize its use in food preservation, thereby reducing the risk of foodborne illnesses. Finally, strain-specific differences in response to citric acid may indicate the presence of inherent genetic or metabolic variations [35], such as differences in the expression of acid-resistant genes or metabolic pathways. A logarithmic period of 8 h was selected for comparison, during which the control group entered the logarithmic phase of bacterial growth, exhibiting a notable increase in growth. At a citric acid concentration of 200 μg/mL, no significant difference was observed between the experimental and control groups (*p* > 0.05). At a citric acid concentration of 400 μg/mL, the growth of VpYZU64, VpYZU68, and VpYZU92 was not significant between the experimental and control groups (*p* > 0.05). In contrast, VpYZU81 and VpYZU110 exhibited significant growth differences compared to the control (*p* < 0.05). At a citric acid concentration of 600 μg/mL, all experimental groups exhibited decreased OD values at 600 nm (*p* < 0.05). At a citric acid concentration of 800 µg/mL, *V. parahaemolyticus* growth was completely inhibited.

### 3.3. Bacteriostatic Effect of the Combination Treatment Phage + Citric Acid in LBS Medium

**Figure 4:**
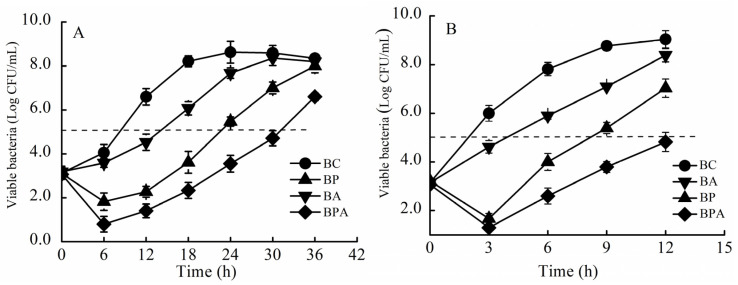
Bacteriostatic efficacy of *VPp*MIx + citric acid in LBS medium. (**A**) 25 °C; (**B**) 37 °C. Abbreviations: BC, blank control group; BP, phage treatment group; BA, citric acid treatment group; BPA, phage + citric acid treatment group.

The inhibitory effect of *VPp*MIx plus 500 μg/mL citric acid on *V. parahaemolyticus* was determined in the culture medium (Figure 4). Table 3 shows the effects of the treatment on bacterial growth reduction. The control, citric acid, phage, and phage + citric acid treatment groups showed a colony count of 10^5^ log CFU/mL at 8, 14, 23, and 31 h, respectively. Citric acid inhibited *V. parahaemolyticus* growth, but its inhibitory effect was lower than that of *VPp*MIx alone. The bacteriostatic efficacy of the *VPp*MIx + citric acid treatment group at 36 h was significantly higher than that of *VPp*MIx alone (*p* < 0.05).

Table 4 shows the effects on bacterial growth. After incubation at 37 °C for 3 h, the total colony count in the control group increased from 3.15 log CFU/mL to 6.0 log CFU/mL. In contrast, the total colony counts in the citric acid, phage, and phage + citric acid treatment groups decreased by 1.38, 4.33, and 4.70 log CFU/mL, respectively. The lowest bacterial count in the phage + citric acid treatment group (1.30 log CFU/mL) was observed at 3 h (*p* < 0.05). After incubation at 37 °C for 9 h, the total colony counts in the citric acid, phage, and phage + citric acid treatment groups decreased by 1.68, 3.38, and 4.98 log CFU/mL, respectively, which were significantly lower than those of the control group (*p* < 0.05). The total colony count increased to 10^5^ log CFU/mL at 2, 4, 8, and 12 h in the control, citric acid, phage, and phage + citric acid treatment groups, respectively. The combination treatment *VPp*MIx + citric acid exhibited better bacterial inhibition in the culture media than citric acid or *VPp*MIx alone.

### 3.4. Bacteriostatic Effect of Phage + Citric Acid in a Salmon Substrate

**Figure 5:**
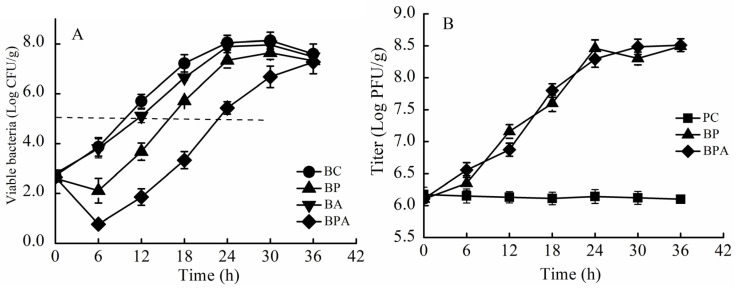
Inhibitory effect of *VPp*MIx + citric acid in salmon fillets incubated at 25 °C. (**A**) Bacterial concentration. (**B**) Phage titer. Abbreviations: BC, blank control group; PC, phage control group; BP, phage treatment group; BA, citric acid treatment group; BPA, phage + citric acid treatment group.

Next, *VPp*MIx was inoculated in salmon fillets under simulated contamination conditions at 25 °C (Figure 5A). Table 5 shows the effects on bacterial growth. Compared with that in the control group, the total *V. parahaemolyticus* count in fillets of the phage treatment group decreased by 1.76 and 2.02 log CFU/g at 6 and 12 h, respectively. The total *V. parahaemolyticus* count in the phage + citric acid treatment group decreased by 3.10 and 3.84 log CFU/g at 6 and 12 h, respectively. In the phage and phage + citric acid treatment groups, the total colony count reached 10^5^ CFU/g at 16 and 23 h, respectively. The bacteriostatic efficacy of citric acid in salmon fillets was not significant. Phage treatment had an inhibitory effect on *V. parahaemolyticus* growth that was significant but less than that of treatment with phage + citric acid.

Phage assays on salmon fillets at 25 °C showed no significant change in phage titer in the phage control group at 24 h (Figure 5B), indicating that the phages maintained stable activity in salmon substrates. The phage titer increased by approximately 2.38 log PFU/g in salmon fillets in the phage and phage + citric acid treatment groups.

**Figure 6:**
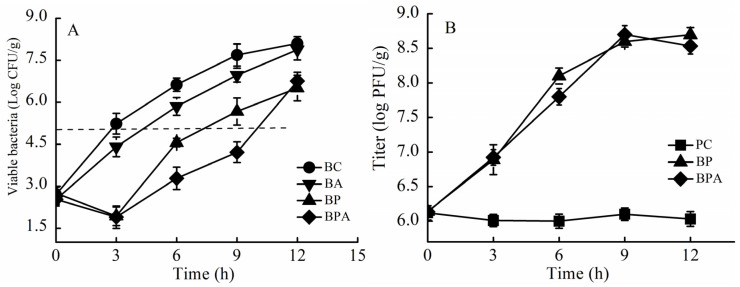
Inhibitory effect of *VPp*MIx + citric acid in salmon at 37 °C. (**A**) Bacterial concentration. (**B**) phage titer. Abbreviations: BC, blank control group; PC, phage control group; BP, phage treatment group; BA, citric acid treatment group; BPA, phage + citric acid treatment group.

The bacteriostatic efficacy of treatments in salmon fillets contaminated with a bacterial suspension of *V. parahaemolyticus* at 37 °C is shown in Figure 6A. Table 6 shows the effects on bacterial growth. The total *V. parahaemolyticus* count in the control group increased to 5.23 log CFU/g after 6 h of storage. Compared to that in the control group, the total *V. parahaemolyticus* count in the citric acid treatment group decreased by 0.83 and 0.78 log CFU/g after 3 and 6 h, respectively (*p* > 0.05); the total *V. parahaemolyticus* count in the phage treatment group decreased by 3.31 and 2.07 log CFU/g, respectively (*p* < 0.05); and the total *V. parahaemolyticus* count in the phage + citric acid treatment group decreased by 3.34 and 3.35 log CFU/g, respectively (*p* < 0.05). The phages detected at 37 °C in each test group are shown in Figure 6B. Salmon fillets were stored for 12 h, and the phage titer in the phage and phage + citric acid treatment groups increased by 2.55 and 2.38 log PFU/g, respectively.

The bacteriostatic effects of phage and citric acid are of significant practical importance for enhancing food safety. Collectively, our results offer a viable approach for managing *V. parahaemolyticus* contamination in food processing environments. Citric acid is a widely used food-grade organic acid, while phage is highly specific for host bacteria. The combination of the two provides a synergistic approach to the control of *V. parahaemolyticus*, which not only inhibits the growth of *V. parahaemolyticus* but also reduces the likelihood of the emergence of drug resistance. Moreover, the capacity of citric acid to disrupt bacterial biofilms, in conjunction with the targeted lysis activity of phages, enables its application in the cleansing and disinfection of food processing equipment and utensils, thus reducing the likelihood of contamination. Incorporating this combination into seafood preservation systems (e.g., marinades or washing solutions) offers an effective means of controlling *V. parahaemolyticus* during food processing and storage. Moreover, implementing this methodology can potentially prolong the shelf life of seafood products while concurrently reducing the likelihood of foodborne illness outbreaks caused by *V. parahaemolyticus*.

Mahmoud [36] found that citric acid effectively inhibited three strains of *V. parahaemolyticus* at a minimum inhibitory concentration of 5.0 mg/mL. After immersing *V. parahaemolyticus*-contaminated oyster meat in 20 mg/mL and 50 mg/mL citric acid solutions for 10 min, the *V. parahaemolyticus* count in the oysters decreased by 0.90 log CFU/g (*p* > 0.05) and 1.80 log CFU/g (*p* < 0.05), respectively. In the present study, *VPp*MIx was combined with 600 μg/mL citric acid as the biological control agent. A relatively low dose of citric acid was used to maintain the sensory quality of salmon fillets. Salmon fillets contaminated with *V. parahaemolyticus* were treated with citric acid for 6 h at 25 °C. The total *V. parahaemolyticus* count decreased by 3.10 log CFU/g (*p* < 0.05), which was higher than that reported previously [36]. This may be due to differences among strains, differences among artificially contaminated substrates, and the improved inhibition of *V. parahaemolyticus* due to the addition of phages.

### 3.5. Biofilm Clearance After Phage + Citric Acid Treatment

**Figure 7:**
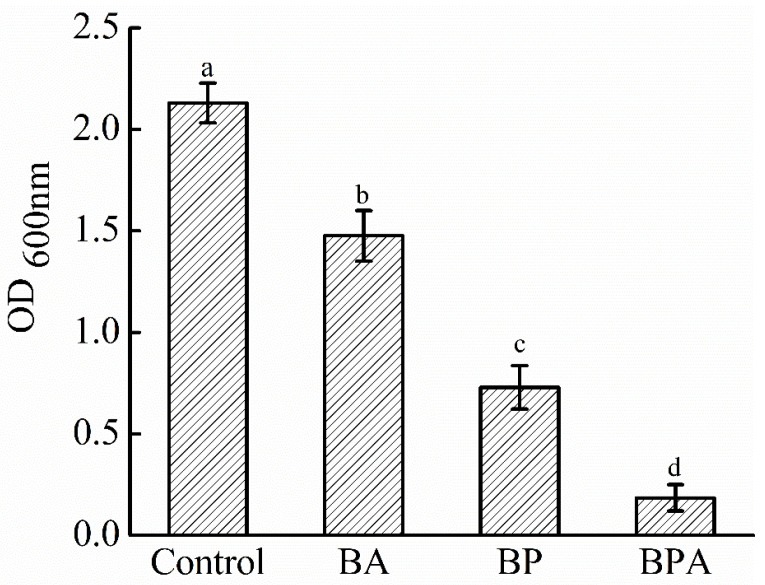
Biofilm clearance after phage + citric acid treatment. Abbreviations: BA, citric acid treatment group; BP, phage treatment group; BPA, phage+citric acid treatment group. Different letters indicate significant differences (*p* < 0.05) among the groups compared.

The formation of biofilms by *V. parahaemolyticus* represents a significant hazard, as these structures serve as reservoirs for pathogens, thereby increasing resistance to cleaning agents and facilitating persistent cross-contamination during food processing. We then observed the clearance of VpYZU64 biofilms after treatment with phage VPpYZU64 (10^9^ PFU/mL) and 600 μg/mL citric acid (Figure 7). Citric acid treatment decreased the OD of host bacteria from 2.13 to 1.48, phage treatment decreased the OD from 2.13 to 0.73, and phage + citric acid treatment decreased the OD from 2.13 to 0.19. Specifically, biofilm reduction after phage + citric acid treatment was highly significant (*p* < 0.05). Removing biofilms is paramount in disrupting the contamination cycle, minimizing the transfer of pathogens to food products, and enhancing food safety [37]. Furthermore, reducing biofilms enhances the efficacy of cleaning and sanitization protocols, thereby mitigating the risk of recurrent contamination and foodborne illnesses.

### 3.6. Observation of Biofilm Clearance via FESEM

**Figure 8:**
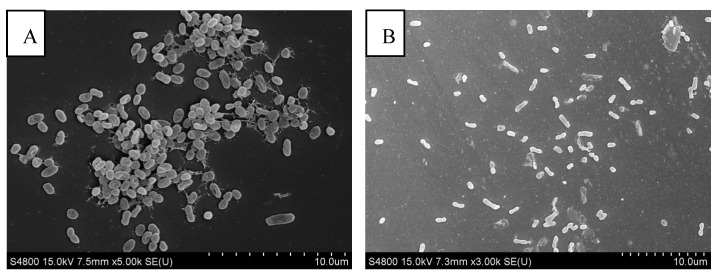

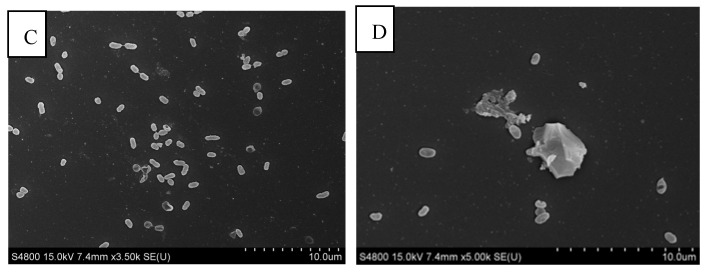
Scanning electron microscopy of biofilms treated with phage VPpYZU64+citric acid for 6 h. (**A**) Control group; (**B**) citric acid treatment group; (**C**) phage treatment group; (**D**) phage+citric acid treatment group.

FESEM observations showed that many intact *V. parahaemolyticus* cells remained in the control group grown at 37 °C for 24 h (Figure 8). At 6 h, the citric acid (Figure 8B) and VPpYZU64 (Figure 8C) treatment groups exhibited some disruption of the cellular structures. The number of cells in the citric acid-treated group was greater than that in the phage-treated group. The VPpYZU64+citric acid treatment group exhibited few bacteria with wrinkled cell surfaces, the formation of intracellular vacuoles, and an inability to form biofilms (Figure 8D). These results show that phages and citric acid inhibit cell membrane formation in *V. parahaemolyticus* and that this effect is more pronounced after treatment with both phages and citric acid than with either phages or citric acid alone.

## 4. Conclusions

This study showed that a phage cocktail + 500 μg/mL citric acid is an effective bacteriostatic agent for controlling *V. parahaemolyticus* in seafood. Moreover, it has potential applications in preventing the proliferation of *V. parahaemolyticus* and the formation of *V. parahaemolyticus* biofilms, particularly under fluctuating temperatures.

However, the research presented here is limited by its small sample size, which could restrict the generalizability of the findings. Additionally, further assessments are necessary to understand how the findings hold under various environmental and processing scenarios. Building on these results, future studies should aim to scale this method for use in industrial settings and to evaluate its long-term effectiveness and safety. Despite these limitations, the study lays the groundwork for the incorporation of phage and citric acid treatments into food safety measures, which could lead to better management of *V. parahaemolyticus* in the seafood industry during both processing and storage.

The transition of phages to commercial biocontrol is confronted with several challenges, including safety, efficacy, resistance, production, and consumer acceptance of “edible viruses.” Notwithstanding these considerations, our study lends support to the use of a phage-citric acid treatment as a means of enhancing the safety of seafood products and managing the contamination of V. parahaemolyticus during the processing and storage phases.

## Figures and Tables

**Table 1:** Statistical data of the predicted gene composition of the phages investigated in this study.

Sample ID	Genome Size (bp)	GC Content (%)	Number of Genes	Gene Total Length (bp)	Gene Average Length (bp)	Gene Length/Genome (%)	GenBank Accession Number
VPpYZU64	76,153	48.92	100	66,396	663.96	87.19	PQ581932
VPpYZU68	58,495	46.54	27	18,868	698.81	32.26	PQ581933
VPpYZU81	42,897	49.28	40	36,600	915.00	85.32	PQ581935
VPpYZU92	80,670	45.04	114	74,577	654.18	92.45	PQ581934
VPpYZU110	144,768	22.51	35	32,592	931.20	22.51	PQ581936

**Table 2:** Classification of phage gene annotation.

Functional Groups of Genes	Number of the Predicted ORFs				
	VPpYZU64	VPpYZU68	VPpYZU81	VPpYZU92	VPpYZU110
Hypothetical	52	12	12	87	1
Structure/morphogenesis	19	5	9	8	11
DNA replication/modification/regulation	17	5	11	8	12
host lysis	1	1	1	1	3
Life activity-related proteins	11	4	7	10	8
Percentage of known functional genes	48.00%	55.56%	70.00%	23.68%	97.14%

**Table 3:** Reduction in *V. parahaemolyticus* levels in LBS medium following treatment with citric acid alone, phage alone, or a combination of citric acid and phage at 25 °C.

Treatment Time (h)	Reduction (log CFU/mL)		
	Citric Acid	PHAGE	Phage + Citric Acid
6	0.47 * ± 0.20 ^c^	2.23 ± 0.39 ^b^	3.26 ± 0.34 ^a^
12	2.07 ± 0.37 ^c^	4.32 ± 0.25 ^b^	5.19 ± 0.31 ^a^
18	2.14 ± 0.30 ^c^	4.60 ± 0.50 ^b^	5.88 ± 0.36 ^a^
24	0.95 ± 0.24 ^c^	3.13 ± 0.17 ^b^	5.08 ± 0.38 ^a^

* Different letters within the same row (lowercase) indicate significant differences (*p* < 0.05).

**Table 4:** Reduction in the levels of *Vp*MIx in LBS medium following treatment with citric acid alone, phage alone, or a combination of citric acid and phage at 37 °C.

Treatment Time (h)	Reduction (log CFU/mL)		
	Citric Acid	Phage	Phage + Citric Acid
3	1.38 * ± 0.26 ^b^	4.33 ± 0.22 ^a^	4.70 ± 0.28 ^a^
6	1.92 ± 0.15 ^c^	3.82 ± 0.35 ^b^	5.22 ± 0.33 ^a^
9	1.68 ± 0.05 ^c^	3.38 ± 0.23 ^b^	4.98 ± 0.23 ^a^
12	0.65 ± 0.27 ^c^	2.00 ± 0.38 ^b^	4.22 ± 0.39 ^a^

* Different letters within the same row (lowercase) indicate significant differences (*p* < 0.05).

**Table 5:** Reduction in the levels of *Vp*MIx in a salmon substrate following treatment with citric acid alone, phage alone, or a combination of citric acid and phage at 25 °C.

Treatment Time (h)	Reduction (log CFU/mL)		
	Citric Acid	Phage	Phage + Citric Acid
6	0.06 * ± 0.38 ^c^	1.76 ± 0.50 ^b^	3.10 ± 0.08 ^a^
12	0.59 ± 0.27 ^c^	2.02 ± 0.35 ^b^	3.84 ± 0.33 ^a^
18	0.57 ± 0.11 ^c^	1.51 ± 0.05 ^b^	3.88 ± 0.34 ^a^
24	0.16 ± 0.44 ^b^	0.71 ± 0.31 ^b^	2.62 ± 0.25 ^a^

* Different letters within the same row (lowercase) indicate significant differences (*p* < 0.05).

**Table 6:** Reduction in the levels of *V. parahaemolyticus* in salmon substrates following treatment with citric acid alone, phage alone, or a combination of citric acid and phage at 37 °C.

Treatment Time (h)	Reduction (log CFU/mL)		
	Citric Acid	Phage	Phage + Citric Acid
3	0.83 * ± 0.35 ^b^	3.31 ± 0.33 ^a^	3.34 ± 0.40 ^a^
6	0.78 ± 0.32 ^c^	2.07 ± 0.15 ^b^	3.35 ± 0.40 ^a^
9	0.72 ± 0.25 ^c^	2.02 ± 0.48 ^b^	3.48 ± 0.37 ^a^
12	0.23 ± 0.37 ^b^	1.59 ± 0.46 ^a^	1.34 ± 0.32 ^a^

* Different letters within the same row (lowercase) indicate significant differences (*p* < 0.05).

## Data Availability

The original contributions presented in this study are included in the article. Further inquiries can be directed to the corresponding author.

## Supplementary Materials

The following supporting information can be downloaded at https://www.mdpi.com/article/10.3390/foods14010037/s1. Figure S1: Genome patterns of the phages investigated in this study. Figure S2: Statistical graph of the correlation between GC content and sequencing depth (Depth) of the five phage strains investigated in this study. Table S2: General features of the putative ORFs identified in VPpYZU64. Table S3: General features of the putative ORFs identified in VPpYZU68. Table S4: General features of the putative ORFs identified in VPpYZU81. Table S5: General features of the putative ORFs identified in VPpYZU92. Table S6: General features of the putative ORFs identified in VPpYZU110.
