# Supplementary material for: Control of Vibrio parahaemolyticus in Seafood Using the Combination of Lytic Phages and Citric Acid
Source: Foods. 2024 Dec 26;14(1):37. doi: 10.3390/foods14010037 (PMC11719698; doi:10.3390/foods14010037)
Supplement: Supplementary file 1 [file foods-14-00037-s001.zip › foods-3359635-supplementary.pdf]

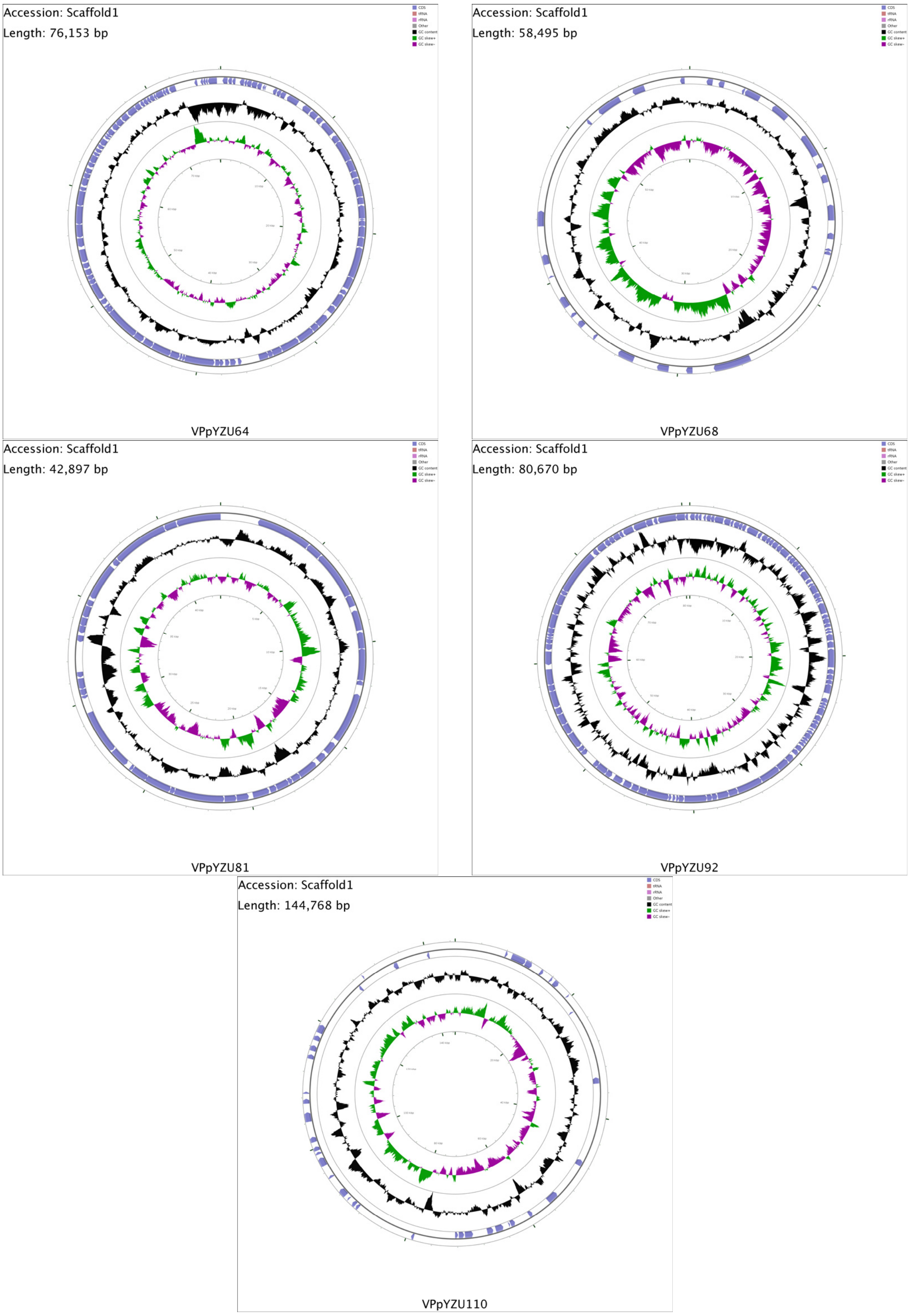

Supplement Figure S1. Genome pattern of the phages investigated in this study. Each circle displays the following (from the outside to the inside): (1) open reading frames (ORFs) transcribed in the clockwise or the counterclockwise direction, (2) G+C % content (values > average are represented by outward peaks, and smaller values are represented by inward peaks), (3) GC skew (G−C/G+C in a 1-kb window and 0.1-kb incremental shift; values greater than zero are in magenta, smaller values are in green), and (4) physical map scaled in Kbp. (To interpret the references to color in this figure legend, the reader is referred to the web version of this article.)

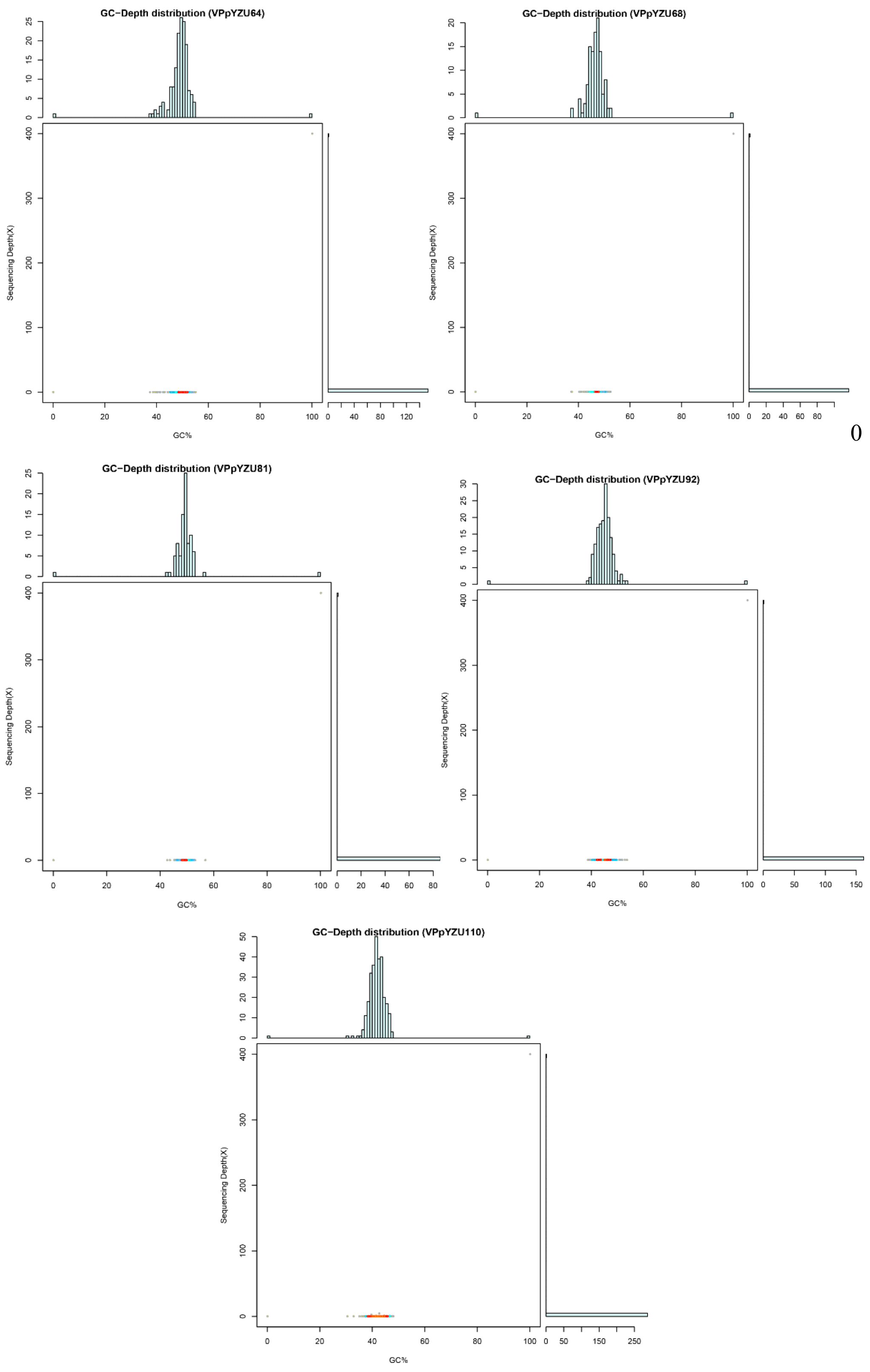

Supplement Figure S2. Statistical graph of the correlation between GC content and sequencing depth (Depth) of the five phage strains investigated in this study. Horizontal coordinates indicate GC content, vertical coordinates indicate sequencing depth, and the distribution of sequencing depth is on the right, and the distribution of GC content is on the top.

Supplement Table S1. Statistics of second-generation sequencing data

Sample ID	Insert size(bp)	Raw data(Mb)	Clean data(Mb)	Reads length(bp)	Clean data GC(%)	Clean data Q20(%)	Clean data Q30(%)
VPpYZU64	500	1,230	1,059	(150:150)	49.21	96.36	90.93
VPpYZU68	500	1,184	1,017	(150:150)	46.53	96.60	91.50
VPpYZU81	500	1,450	1,197	(150:150)	49.53	96.77	91.76
VPpYZU92	500	1,115	947	(150:150)	45.41	96.69	91.73
VPpYZU110	500	971	820	(150:150)	42.07	95.87	90.16

Supplement Table S2. General features of the putative ORFs identified in VPpYZU64

ORFs	Start	End	Strand	Length	Putative function	Best phage homolog(Identity)	Accession no.	E_value
1	153	590	-	438	hypothetical protein	Vibrio phage VVP001(100.00%)	AUM58706.1	4.00E-102
2	640	963	-	324	hypothetical protein	Vibrio phage VP06(98.15%)	AVI05193.1	3.00E-73
3	995	1270	-	276	hypothetical protein	Vibrio phage vB_VpaS_KF6(100.00%)	ATI19498.1	3.00E-61
4	1643	2017	-	375	hypothetical protein	Vibrio phage vB_VneS_S3(98.40%)	XGU05059.1	4.00E-86

5	2034	2258	-	225	hypothetical protein	Vibrio phage vB_VpS_C2(99.95%)	QYW05990.1	2.00E-45
6	2251	2652	-	402	hypothetical protein	Vibrio phage vB_VpS_C2(99.25%)	QYW05989.1	5.00E-93
7	2658	3143	-	486	hypothetical protein	Vibrio phage vB_VneS_S3(99.38%)	XGU05157.1	3.00E-115
8	3161	3550	-	390	hypothetical protein	Vibrio phage vB_VpaS_KF3(99.23 %)	ATI19169.1	1.00E-87
9	3654	3803	-	150	hypothetical protein	Vibrio phage vB_VpaS_KF3(100.0 0%)	ATI19168.1	1.00E-26
10	3803	3928	-	126	hypothetical protein	Vibrio phage SSP002(97.62%)	YP_009598679.1	8.00E-21
11	4602	4919	-	318	TM2 domain-containing protein	Vibrio alginolyticus(100.00%)	WP_217846552.1	1.00E-71
12	4952	:5287	-	336	hypothetical protein	Vibrio alginolyticus(99.11%)	WP_217846554.1	3.00E-76
13	5284	5742	-	459	hypothetical protein	Vibrio alginolyticus(99.35%)	WP_217846556.1	3.00E-109
14	5945	6988	-	1044	ParB N-terminal domain-containing protein	Vibrio alginolyticus(100.00%)	WP_217846559.1	0.00E+00

15	7524	8240	-	717	hypothetical protein	Vibrio phage VVP001(100.00%)	AUM58719.1	1e-171
16	8316	8999	-	684	hypothetical protein	Vibrio alginolyticus(100.00%)	WP_217846564.1	4.00E-167
17	9095	9586	-	492	hypothetical protein	Vibrio phage VPy01(100.00%)	WJZ44406.1	4.00E-117
18	9733	10914	-	1182	putative pyrophosphatase	Vibrio phage vB_ValS_R12Z(100.00%)	WVW36981.1	0.00E+00
19	11142	11756	-	615	putative 6-pyruvoyl tetrahydropterin synthase	Vibrio phage F23s1(99.02%)	UCW44074.1	2.00E-147
20	11761	13605	-	1845	putative exonuclease	Vibrio phage VPy01(99.02%)	WJZ44409.1	0.00E+00
21	13608	14594	-	987	metallophosphoesterase	Vibrio alginolyticus(100.00%)	WP_217847357.1	0.00E+00
22	14594	15181	-	588	holliday junction DNA helicase	Vibrio phage vB_VpaS_KF6(100.00%)	ATI19480.1	1.00E-140
23	15234	15758	-	525	hypothetical protein	Vibrio phage VVP001(100.00%)	AUM58727.1	2.00E-128
24	15775	16875	-	1101	ATPase domain-containing protein	Vibrio alginolyticus(100.00%)	WP_217846576.1	0.00E+00

25	16906	17538	-	633	putative thymidylate kinase	Vibrio phage VVP001(100.00%)	AUM58729.1	3.00E-154
26	17621	18757	-	1137	single-stranded DNA-binding domain protein	Vibrio phage VPy01(99.47%)	WJZ44413.1	0.00E+00
27	18763	18900	-	138	hypothetical protein PG288_34	Vibrio phage PG288(100.00%)	WGL39714.1	4.00E-24
28	18905	19393	-	489	hypothetical protein	Vibrio alginolyticus(100.00%)	WP_217846583.1	2.00E-113
29	19400	20332	-	933	putative DNA polymerase III subunit	Vibrio phage VVP001	AUM58733.1	0.00E+00
30	20350	21198	-	849	thymidylate synthase	Vibrio phage vB_VpaS_KF5(100.00%)	ATI19378.1	0.00E+00
31	21250	23901	-	2652	DNA polymerase	Vibrio alginolyticus(100.00%)	WP_217846586.1	0.00E+00
32	23907	24965	-	1059	DNA polymerase processivity factor	Vibrio phage 31Fb.4(99.43%)	UYE96286.1	0.00E+00
33	25131	25529	-	399	hypothetical protein	Vibrio alginolyticus(100.00%)	WP_217878206.1	3.00E-95
34	25611	26762	-	1152	hypothetical protein	Vibrio phage VVP001(100.00%)	AUM58738.1	0.00E+00

35	26837	27451	-	615	putative RNA polymerase sigma factor	Vibrio phage vB_VpS_CC6(99.02%)	UTQ72795.1	4.00E-151
36	27871	28923	-	1053	DNA primase	Vibrio phage vB_VpaS_KF5(99.72%)	ATI19372.1	0.00E+00
37	28923	29537	-	615	hypothetical protein	Staphylococcus argenteus(100.00%)	MCG9825314.1	7.00E-150
38	29541	30989	-	1449	helicase	Vibrio phage vB_ValS_R12Z(100.00%)	WVW36961.1	0.00E+00
39	31054	32661	-	1608	DnaB-like helicase C-terminal domain-containing protein	Vibrio alginolyticus(100.00%)	WP_217862573.1	0.00E+00
40	32664	33515	-	852	putative DNA polymerase I	Vibrio phage VVP001(100.00%)	AUM58744.1	0.00E+00
41	33502	33921	-	420	hypothetical protein	Vibrio phage VVP001(100.00%)	AUM58745.1	6.00E-98
42	33908	34741	-	834	DnaC-like helicase loader	Vibrio phage SSP002(100.00%)	YP_009598647.1	0.00E+00
43	36270	36578	-	309	hypothetical protein	Vibrio phage R01(76.29%)	AXH68419.1	1.00E-47
44	36764	37252	-	489	hypothetical protein	Vibrio phage SSP002(100.00%)	YP_009598644.1	1.00E-110

45	37311	37685	-	375	endolysin-like protein	Vibrio phage OTA22(100.00%)	UGC97207.1	5.00E-87
46	37825	38136	-	312	hypothetical protein	Vibrio phage SSP002(100.00%)	YP_009598642.1	3.00E-66
47	38117	38536	-	420	hypothetical protein	Vibrio alginolyticus(100.00%)	WP_217862580.1	2.00E-93
48	38598	41150	-	2553	putative tail protein	Vibrio phage VVP001(99.29%)	AUM58754.1	0.00E+00
49	41134	41376	-	243	tail assembly chaperone	Vibrio phage SSP002(98.77%)	YP_009598639.1	2.00E-52
50	41379	41618	-	240	tail assembly chaperone	Vibrio phage SSP002(100.00%)	YP_009598638.1	1.00E-50
51	41642	42445	-	804	putative tail assembly protein	Vibrio phage ValLY_3(97.39%)	QAY01769.1	0.00E+00
52	42448	44115	-	1668	putative tail assembly protein	Vibrio phage OTA22(93.02%)	UGC97199.1	0.00E+00
53	44130	44993	-	864	virion protein	Vibrio phage VPy01(65.40%)	WJZ44433.1	4.00E-136
54	45080	45910	-	831	virion protein	Vibrio phage vB_VpS_C2(90.25%)	QYW05940.1	0.00E+00
55	45916	48759	-	2844	putative tail tape measure protein	Vibrio phage 13KS502A(99.37%)	WGH28626.1	0.00E+00
56	48762	49214	-	453	tail completion or Neck1 protein	Vibrio phage SSP002(100.00%)	YP_009598632.1	5.00E-107

57	49192	49437	-	246	tail chaperoninne	Vibrio phage VPy01(100.00%)	WJZ44435.1	7.00E-52
58	49584	50033	-	450	putative structural protein	Vibrio phage vB_VneS_S3(81.21%)	XGU05105.1	9.00E-85
59	50127	51566	-	1440	major tail tube protein	Vibrio phage VPy01(99.79%)	WJZ44437.1	0.00E+00
60	51581	51991	-	411	phage tail terminator-like protein	Vibrio alginolyticus(100.00%)	WP_217846660.1	7.00E-98
61	51991	52383	-	393	hypothetical protein	Vibrio phage vB_VpaS_KF6(100.00%)	ATI19439.1	6.00E-93
62	52399	52938	-	540	virion structural protein	Vibrio phage VPy01(100.00%)	WJZ44439.1	3.00E-131
63	53026	53493	-	468	hypothetical protein	Vibrio alginolyticus(99.36%)	WP_217846671.1	2.00E-108
64	53573	54697	-	1125	P22 phage major capsid protein family protein	Vibrio alginolyticus(100.00%)	WP_217847368.1	0.00E+00
65	54909	55697	-	789	putative peptidase	Vibrio phage vB_VpaS_KF5(98.48%)	ATI19341.1	0.00E+00
66	55746	56861	-	1116	phage minor head protein	Vibrio alginolyticus(99.73%)	WP_217846677.1	0.00E+00

67	56868	58433	-	1566	putative structural protein	Vibrio phage vB_VpS_CC6(100.00%)	UTQ72761.1	0.00E+00
68	58450	59751	-	1302	terminase large subunit domain-containing protein	Vibrio alginolyticus(100.00%)	WP_217846681.1	0.00E+00
69	59774	60115	-	342	HNH endonuclease	Vibrio alginolyticus(100.00%)	WP_217846683.1	2.00E-79
70	60228	60875	-	648	hypothetical protein	Vibrio phage 27Ua.3(100.00%)	UYD21430.1	2.00E-157
71	60871	61323	-	453	hypothetical protein	Vibrio alginolyticus(100.00%)	WP_217846685.1	4.00E-108
72	61328	61771	-	444	putative NAD-dependent DNA ligase protein	Vibrio phage PG288(98.65%)	WGL39761.1	1.00E-101
73	61909	62304	-	396	hypothetical protein	Vibrio alginolyticus(99.24%)	WP_217847373.1	3.00E-92
74	62312	62962	-	651	conjugal transfer protein	Vibrio phage vB_VpaS_KF6(99.54%)	ATI19426.1	1.00E-162
75	62976	63269	-	294	conjugal transfer protein	Vibrio phage vB_VpS_CC6(96.94%)	UTQ72752.1	1.00E-62

76	63304	63633	-	330	hypothetical protein	Vibrio phage vB_ValS_R12Z(100.00%)	WVW36921.1	2.00E-75
77	63703	64029	-	327	hypothetical protein	Vibrio phage VPy01(100.00%)	WJZ44451.1	8.00E-76
78	64060	64605	-	546	PadR family transcriptional regulator	Vibrio alginolyticus(100.00%)	WP_217846697.1	8.00E-134
79	64712	64936	-	225	hypothetical protein	Vibrio phage vB_ValS_R12Z(100.00%)	WVW36918.1	3.00E-45
80	64939	65157	-	219	hypothetical protein	Vibrio alginolyticus(98.63%)	WP_217846699.1	2.00E-46
81	65157	65636	-	480	hypothetical protein	Vibrio phage SSP002(98.12%)	YP_009598607.1	9.00E-115
82	65744	66229	-	486	BRCT domain-containing protein	Vibrio alginolyticus(99.38%)	WP_217846703.1	2.00E-113
83	66232	66801	-	570	hypothetical protein	Vibrio alginolyticus(100.00%)	WP_217846705.1	7.00E-137
84	66850	67461	-	612	hypothetical protein	Vibrio phage ValLY_3(98.53%)	QAY01801.1	2.00E-145
85	67551	68330	-	780	DUF2829 domain-containing protein	Vibrio phage SSP002(99.62%)	YP_009598603.1	0.00E+00

86	68333	68833	-	501	hypothetical protein	Vibrio phage vB_VpaS_KF5(98.20%)	ATI19321.1	7.00E-114
87	68891	69184	-	294	hypothetical protein	Vibrio alginolyticus(95.92%)	WP_217878216.1	7.00E-65
88	69197	69541	-	345	hypothetical protein	Vibrio phage SSP002(100.00%)	YP_009598600.1	1.00E-77
89	69541	69813	-	273	hypothetical protein	Vibrio phage ValLY_3(100.00%)	QAY01806.1	1.00E-61
90	69818	70195	-	378	hypothetical protein	Vibrio phage vB_ValS_R12Z(99.21%)	WVW36907.1	2.00E-87
91	70203	70397	-	195	hypothetical protein	Vibrio alginolyticus(100.00%)	WP_217847381.1	2.00E-40
92	70390	71055	-	666	NADH-PPase NADH pyrophosphatase zinc ribbon domain protein	Vibrio phage vB_VpS_C2(98.20%)	QYW05901.1	1.00E-161
93	71060	71281	-	222	hypothetical protein	Vibrio phage vB_VpaS_HCMJ(98.65%)	QEP53468.1	6.00E-46
94	71286	71579	-	294	hypothetical protein	Vibrio alginolyticus(100.00%)	WP_217846727.1	1.00E-66

95	71657	72220	-	564	HNH endonuclease	Vibrio phage vB_VpS_C2(99.47%)	QYW05897.1	1.00E-136
96	73891	74139	-	249	hypothetical protein	Vibrio phage vB_VpS_C2(99.95%)	QYW06000.1	2.00E-54
97	74419	74631	-	213	hypothetical protein	Vibrio phage SSP002(100.00%)	YP_009598692.1	8.00E-44
98	74636	74854	-	219	hypothetical protein	Vibrio phage vB_ValS_R12Z(98.63 %)	WVW37004.1	1.00E-44
99	74859	75038	-	180	hypothetical protein	Vibrio alginolyticus(98.33%)	WP_217878163.1	4.00E-35
100	75056	75832	-	777	hypothetical protein	Vibrio phage vB_VpaS_KF3(99.23 %)	ATI19177.1	0.00E+00

Supplement Table S3. General features of the putative ORFs identified in VPpYZU68

ORFs	Start	End	Strand	Length	Putative function	Best phage homolog(Identity)	Accession no.	E_value
1	1102	1509	-	408	hypothetical protein	Vibrio phage VspDsh_1(100.00%)	QAY02065.1	1.00E-95
2	1906	2283	-	378	hypothetical protein	Vibrio phage vB_VpS_CA8(100.00%)	QEA10932.1	2.00E-85
3	3517	3687	-	171	hypothetical protein	Vibrio phage vB_VpS_CA8(100.00%)	QEA10929.1	4.00E-30
4	3737	4783	-	1047	capsid protein	Vibrio phage PH669(99.71%)	QQK88517.1	0
5	5825	6985	-	1161	minor head protein-like protein	Vibrio phage VspDsh_1 (99.74%)	QAY02072.1	0
6	8565	10130	-	1566	terminase large subunit	Vibrio phage VspDsh_1(100.00%)	QAY02074.1	0
7	10656	10979	-	324	holin	Vibrio phage vB_VpS_CA8(98.15%)	QEA10921.1	2.00E-67
8	11470	12009	-	540	TerS	Vibrio phage VspDsh_1 (100.00%)	QAY02078.1	3.00E-129
9	13506	14543	-	1038	DNA ligase	Vibrio phage vB_VpaS_AL-2(99.42%)	UFK26996.1	0

10	15351	15767	-	417	hypothetical protein	Enterobacteria phage JenP2(33.33%)	AKA61032.1	7.42E-09
11	16400	16573	-	174	hypothetical protein	Vibrio phage vB_VpaS_AL-2(91.38%)	UFK27001.1	1.00E-28
12	16587	16829	-	243	hypothetical protein	Vibrio phage vB_VpaS_AL-2(96.30%)	UFK27002.1	2.00E-51
13	19041	19232	-	192	hypothetical protein	Streptococcus suis(98.41%)	WP_203200717.1	1.00E-36
14	25377	27749	+	2373	primase	Vibrio phage VspDsh_1(96.71%)	QAY02025.1	0
15	29079	29486	+	408	hypothetical protein	Paracoccus phage vB_PmaS_IMEP1(34.62%)	YP_009126401.1	8.73E-13
16	30574	31314	+	741	ATPase	Vibrio phage VspDsh_1 (100.00%)	QAY02031.1	0
17	32844	33887	+	1044	exonuclease	Vibrio phage vB_VpS_CA8 (95.69%%)	QEA10967.1	0
18	35579	35764	+	186	hypothetical protein	Vibrio phage VspDsh_1(98.39%)	QAY02036.1	9.00E-39
19	36650	37700	+	1051	hypothetical protein	Vibrio phage VspDsh_1(70.10%)	QAY02039.1	7.00E-88
20	38207	38908	+	702	orgamic radical activating enzyme	Vibrio phage VspDsh_1(100.00%)	QAY02041.1	2.00E-173

21	40299	40412	+	114	hypothetical protein	Vibrio phage VspDsh_1(81.08%)	QAY02043.1	1.00E-13
22	41159	41788	+	630	putative GTP cyclohydrolase	Vibrio phage VspDsh_1(99.50%)	QAY02045.1	2.00E-147
23	43578	44549	+	972	DNA polymerase beta subunit	Vibrio phage vB_VpS_CA8(100.00%)	QEA10951.1	0
24	51003	51215	-	213	tail assembly protein	Vibrio phage vB_VpS_CA8 (100.00%)	QEA10942.1	9.00E-43
25	52030	53673	-	1644	tail assembly protein	Vibrio phage vB_VpS_CA8(89.40%)	QEA10940.1	0
26	54629	55459	-	831	virion protein	Vibrio phage vB_VpS_C2 (72.20%)	QYW05940.1	8.00E-149
27	57854	58153	-	300	hypothetical protein	Pseudomonas phage PaMx11, PaMx11_24(40%)	ALH23698.1	2.85E-17

Supplement Table S4. General features of the putative ORFs identified in VPpYZU81

ORFs	Start	End	Strand	Length	Putative function	Best phage homolog(Identity)	Accession no.	E_value
1	1828	4503	-	2676	internal virion protein	Vibrio phage vB_VpP_HA1(99.33%)	UXF57315.1	0
2	4516	5259	-	744	internal virion protein	Vibrio phage vB_VpP_FE11(88.71%)	QIW87180.1	3.00E-146
3	5275	7614	-	2340	putative tail tubular protein B	Vibrio phage F23s2(99.62%)	UPT53618.1	0
4	7627	8184	-	558	putative tail tubular protein A	Vibrio phage vB_VpaP_MGD1 (100.00%)	QKK83137.1	0
5	8393	9388	-	996	major capsid protein	Vibrio phage vB_VpP_DE10(100.00%)	QXV72174.1	0
6	9456	10268	-	813	head scaffolding protein	Vibrio phage VP93(100.00%)	YP_002875652.1	0
7	10271	11800	-	1530	head-tail connector protein	Vibrio phage VP93(100.00%)	YP_002875651.1	0
8	11812	12054	-	243	virion protein	Vibrio phage vB_VpaP_AL-1(98.77%)	UFK26906.1	3.00E-47
9	12495	14942	-	2448	DNA-directed RNA polymerase	Vibrio phage VPy02 (100.00%)	WJZ23406.1	0
10	15131	15712	-	582	putative deoxynucleoside monophosphate kinase	Vibrio phage vB_VpP_HA5(98.97%)	UXF57373.1	7.00E-140
11	15831	16268	-	438	endonuclease	Vibrio phage vB_VpP_DE18(100.00%)	QWY13562.1	1.00E-101
12	16451	17398	-	948	putative exonuclease	Vibrio phage F23s2(100.00%)	UPT53632.1	0

13	17404	17826	-	423	HAMP domain-containing protein	Vibrio phage VPy02 (98.58%)	WJZ23413.1	1.00E-92
14	17839	18039	-	201	hypothetical protein	Vibrio phage VP93(100.00%)	YP_002875644.1	4.00E-41
15	18042	18668	-	627	pyrophosphatase	Vibrio phage VPy02(100.00%)	WJZ23415.1	3.00E-150
16	18683	19012	-	330	hypothetical protein	Vibrio phage F23s2(100.00%)	UPT53636.1	7.00E-76
17	19062	19874	-	813	hypothetical protein	Vibrio phage VP93(100.00%)	YP_002875641.1	0
18	20079	20669	-	591	nucleotidyltransferase	Vibrio phage VP93(98.98%)	YP_002875640.1	1.00E-141
19	20681	21256	-	576	hypothetical protein	Vibrio phage VPy02(99.48%)	WJZ23419.1	3.00E-138
20	21273	23696	-	2424	DNA polymerase	Vibrio OY1(99.26%)	UMW87792.1	0
21	23695	23922	-	228	putative DNA helicase	Vibrio phage vB_VpP_FE11(98.68%)	QIW87157.1	3.00E-49
22	23925	25202	-	1278	putative DNA helicase	Vibrio phage vB_VpaP_KF1(100.00%)	YP_009808050.1	0
23	25187	25996	-	810	putative primase	Vibrio phage VPy02(100.00%)	WJZ23422.1	0
24	25999	26160	-	162	hypothetical protein	Vibrio phage VP93(100.00%)	YP_002875633.1	6.00E-31
25	26393	27292	-	900	metallopeptidase activity protein	Vibrio phage vB_VpP_AC2(98.00%)	UTQ72429.1	0
26	27331	29469	-	2139	hypothetical protein	Vibrio phage F23s2(99.44%)	UPT53647.1	0
27	30175	30321	-	147	hypothetical protein	Vibrio phage TCU_VP01_ZB(97.96%)	WZW26863.1	2.00E-24

28	30334	30642	-	309	helix-turn-helix transcriptional regulator	Vibrio phage vB_VpaS_OWB (100.00%)	YP_009948711.1	5.00E-69
29	30648	31181	-	534	hypothetical protein	Vibrio phage VP93(100.00%)	YP_002875626.1	1.00E-127
30	31234	31494	-	261	hypothetical protein	Vibrio phage vB_VpP_HA5(100.00%)	UXF57396.1	1.00E-56
31	32937	33257	-	321	hypothetical protein	Vibrio phage VP93(63.96%)	YP_002875667.1	7.00E-29
32	33378	33731	-	354	hypothetical protein	Vibrio phage vB_VpaP_KF1(100.00%)	YP_009808039.1	9.00E-81
33	33727	34137	-	411	endolysin	Vibrio phage vB_VpaP_KF1(99.27%)	YP_009808038.1	7.00E-96
34	34162	34716	-	555	bacterial Ig-like domain family protein	Vibrio phage vB_VpP_DE10(98.38%)	QXV72187.1	6.00E-127
35	34728	35024	-	297	hypothetical protein	Vibrio phage vB_VpaP_MGD1(100.00%)	QKK83146.1	2.00E-63
36	35132	36934	-	1803	terminase large subunit	Vibrio phage vB_VpaP_KF2(100.00%)	YP_009808066.1	0
37	37062	37358	-	297	terminase small subunit	Vibrio phage VP93 (100.00%)	YP_002875661.1	5.00E-60
38	37371	40100	-	2730	putative glycosyl hydrolase	Vibrio phage vB_VpaP_KF1(99.45%)	YP_009808032.1	0
39	40112	40720	-	609	tail fiber protein	Vibrio phage vB_VpP_HA5 (100.00%)	UXF57360.1	4.00E-147
40	40744	42897	-	2154	peptidoglycan lytic exotransglycosylase	Vibrio phage vB_VpP_DE18(99.72%)	QWY13576.1	0

Supplement Table S5. General features of the putative ORFs identified in VPpYZU92

ORFs	Start	End	Strand	Length	Putative function	Best phage homolog(Identity)	Accession no.	E_value
1	1	441	-	441	hypothetical protein	Vibrio phage vB_VhaS-VHB1(100%)	QKE60690.1	1.00E-102
2	458	757	-	300	hypothetical protein	Vibrio phage SIO-2(100%)	YP_004957605.1	2.00E-66
3	772	1056	-	285	hypothetical protein	Vibrio phage SIO-2(100%)	YP_004957606.1	8.00E-60
4	1224	1454	-	231	hypothetical protein	Vibrio phage SIO-2(100%)	YP_004957607.1	2.00E-46
5	1499	1903	-	405	hypothetical protein	Vibrio phage vB_VhaS-VHB1(100%)	QKE60803.1	8.00E-94
6	1893	2228	-	336	hypothetical protein	Vibrio phage SIO-2, VPEG_00002(100%)	YP_004957495.1	1.00E-74
7	2237	3061	-	825	ssDNA binding protein	Vibrio phage vB_VhaS-VHB1(100%)	QKE60801.1	0.00E+00
8	3132	3719	-	588	hypothetical protein	Vibrio phage vB_VhaS-VHB1(100%)	QKE60800.1	4.00E-138
9	3734	4000	-	267	hypothetical protein	Vibrio phage vB_VhaS-VHB1(100%)	QKE60799.1	5.00E-55

10	4067	4540	-	474	hypothetical protein	Vibrio phage vB_VhaS-VHB1(100%)	QKE60798.1	5.00E-110
11	4521	4856	-	336	hypothetical protein	Vibrio phage vB_VhaS-VHB1(100%)	QKE60797.1	4.00E-76
12	4861	5211	-	351	hypothetical protein	Vibrio phage vB_VhaS-VHB1(100%)	QKE60796.1	2.00E-78
13	5358	6383	-	1026	hypothetical protein	Vibrio phage vB_VhaS-VHB1(99.42%)	QKE60795.1	0.00E+00
14	6458	6784	-	327	hypothetical protein	Vibrio phage SIO-2(100%)	YP_004957503.1	3.00E-72
15	6888	7364	-	477	hypothetical protein	Vibrio phage vB_VhaS-VHB1(99.37%)	QKE60793.1	1.00E-110
16	7360	7692	-	333	hypothetical protein	Vibrio phage SIO-2(100%)	YP_004957505.1	1.00E-70
17	7683	8024	-	342	hypothetical protein	Vibrio phage vB_VhaS-VHB1(99.12%)	QKE60791.1	5.00E-78
18	8033	8533	-	501	hypothetical protein	Vibrio phage SIO-2(99.40%)	YP_004957507.1	4.00E-121
19	8545	8976	-	432	hypothetical protein	Vibrio phage vB_VhaS-VHB1(100%)	QKE60789.1	7.00E-103
20	8976	9809	-	834	hypothetical protein	Vibrio phage vB_VhaS-VHB1(100%)	QKE60788.1	0.00E+00
21	9905	10168	-	264	hypothetical protein	Vibrio phage SIO-2(100%)	YP_004957510.1	7.00E-57

22	10164	10589	-	426	hypothetical protein	Vibrio phage SIO-2(100%)	YP_004957511.1	6.00E-94
23	10582	11031	-	450	hypothetical protein	Vibrio phage vB_VhaS-VHB1(95.33%)	QKE60785.1	4.00E-103
24	11027	11386	-	360	hypothetical protein	Vibrio phage vB_VhaS-VHB1(100%)	QKE60784.1	4.00E-83
25	11370	11684	-	315	hypothetical protein	Vibrio phage SIO-2(100%)	YP_004957514.1	5.00E-70
26	11771	12556	-	786	hypothetical protein	Vibrio phage vB_VhaS-VHB1(100%)	QKE60782.1	0.00E+00
27	12617	12946	-	330	hypothetical protein	Vibrio phage SIO-2(100%)	YP_004957516.1	1.00E-70
28	13045	13287	-	243	holin	Vibrio phage SIO-2(96.30%)	YP_004957517.1	1.00E-48
29	13287	14054	-	768	hypothetical protein	Vibrio phage Vpas_PP24(97.27%)	UOX38358.1	0.00E+00
30	14067	14516	-	450	hypothetical protein	Vibrio phage SIO-2(100%)	YP_004957519.1	4.00E-106
31	14512	14763	-	252	hypothetical protein	Vibrio phage SIO-2(98.81%)	YP_004957520.1	6.00E-52
32	14750	14950	-	201	hypothetical protein	Vibrio phage vB_VhaS-VHB1(100%)	QKE60776.1	2.00E-40
33	15065	16054	-	990	hypothetical protein	Vibrio phage vB_VhaS-VHB1(99.39%)	QKE60775.1	0.00E+00

34	16141	16494	-	354	hypothetical protein	Vibrio phage SIO-2(100%)	YP_004957523.1	1.00E-79
35	16497	16982	-	486	hypothetical protein	Vibrio phage vB_VhaS-VHB1(100%)	QKE60773.1	3.00E-111
36	16982	17335	-	354	hypothetical protein	Vibrio phage vB_VhaS-VHB1(98.31%)	QKE60772.1	1.00E-77
37	17399	17635	-	237	hypothetical protein	Vibrio phage vB_VhaS-VHB1(100%)	QKE60771.1	3.00E-50
38	17652	17903	-	252	hypothetical protein	Vibrio phage SIO-2(98.81%)	YP_004957527.1	6.00E-50
39	17993	18583	-	591	hypothetical protein	Vibrio phage vB_VhaS-VHB1(99.49%)	QKE60769.1	9.00E-140
40	18651	19244	-	594	hypothetical protein	Vibrio phage vB_VhaS-VHB1(99.49%)	QKE60768.1	1.00E-142
41	19333	21024	-	1692	hypothetical protein	Vibrio phage SIO-2(95.92%)	YP_004957530.1	0.00E+00
42	21160	21615	-	456	hypothetical protein	Vibrio phage SIO-2(97.37%)	YP_004957531.1	1.00E-105
43	21639	22121	-	483	hypothetical protein	Vibrio phage vB_VhaS-VHB1(98.14%)	QKE60764.1	2.00E-112
44	22126	22560	-	435	hypothetical protein	Vibrio phage vB_VhaS-VHB1(98.62%)	QKE60763.1	3.00E-103
45	22566	23414	-	849	hypothetical protein	Vibrio phage Vpas_PP24(96.74%)	UOX38341.1	0.00E+00

46	23865	24266	-	402	hypothetical protein	Streptococcus suis(100%)	WP_203200699.1	6.00E-94
47	24269	24550	-	282	hypothetical protein	Vibrio phage vB_VhaS-VHB1(100%)	QKE60760.1	1.00E-61
48	24553	24807	-	255	hypothetical protein	Vibrio phage SIO-2(100%)	YP_004957537.1	6.00E-53
49	24803	25477	-	675	hypothetical protein	Vibrio phage vB_VhaS-VHB1(99.11%)	QKE60758.1	1.00E-159
50	25545	26039	-	495	hypothetical protein	Vibrio phage vB_VhaS-VHB1(100%)	QKE60757.1	3.00E-115
51	26046	26357	-	312	cell division control protein	Vibrio phage vB_VhaS-VHB1(100%)	QKE60756.1	4.00E-70
52	26347	26811	-	465	hypothetical protein	Vibrio phage Vpas_PP24(83.87%)	UOX38452.1	1.00E-85
53	26873	27055	-	183	hypothetical protein	Vibrio phage vB_VhaS-VHB1(100%)	QKE60754.1	7.00E-35
54	27122	27415	-	294	hypothetical protein	Vibrio phage SIO-2(100%)	YP_004957543.1	1.00E-65
55	27415	27900	-	486	hypothetical protein	Vibrio phage SIO-2(98.77%)	YP_004957544.1	1.00E-112
56	27894	28163	-	270	hypothetical protein	Vibrio phage SIO-2(97.78%)	YP_004957545.1	5.00E-55
57	28163	28423	-	261	hypothetical protein	Vibrio phage vB_VhaS-VHB1(98.85%)	QKE60750.1	2.00E-57

58	28428	28721	-	294	hypothetical protein	Vibrio phage SIO-2(100%)	YP_004957547.1	3.00E-65
59	28800	29261	-	462	hypothetical protein	Vibrio phage vB_VhaS-VHB1(99.35%)	QKE60748.1	2.00E-110
60	29301	29726	-	426	transmembrane helix protein	Vibrio phage vB_VhaS-VHB1(100%)	QKE60747.1	2.00E-96
61	29935	30579	-	645	DNA methyltransferase	Vibrio phage SIO-2(100%)	YP_004957550.1	3.00E-160
62	30590	31660	-	1071	portal protein	Vibrio phage vB_VhaS-VHB1(99.72%)	QKE60745.1	0.00E+00
63	31669	33531	-	1863	capsid portal protein	Vibrio phage SIO-2(99.84%)	YP_004957552.1	0.00E+00
64	33535	35886	-	2352	DNA polymerase	Vibrio phage SIO-2(99.74%)	YP_004957553.1	0.00E+00
65	35901	36752	-	852	pyruvate decarboxylase	Vibrio phage vB_VhaS-VHB1(98.23%)	QKE60742.1	0.00E+00
66	36758	37789	-	1032	hypothetical protein	Vibrio phage SIO-2(99.42%)	YP_004957555.1	0.00E+00
67	37822	38295	-	474	tyrosine phosphatase	Vibrio phage vB_VhaS-VHB1(100%)	QKE60740.1	6.00E-114
68	38291	38770	-	480	tyrosine phosphatase	Vibrio phage vB_VhaS-VHB1(99.38%)	QKE60739.1	8.00E-113
69	38773	40815	-	2043	pyruvate phosphate dikinase	Vibrio phage vB_VhaS-VHB1(99.41%)	QKE60738.1	0.00E+00

70	40849	41328	-	480	hypothetical protein	Vibrio phage vB_VhaS-VHB1(100%)	QKE60737.1	4.00E-111
71	41321	41566	-	246	hypothetical protein	Vibrio phage vB_VhaS-VHB1(100%)	QKE60736.1	6.00E-52
72	41630	41896	-	267	hypothetical protein	Vibrio phage vB_VhaS-VHB1(100%)	QKE60735.1	9.00E-58
73	41997	42242	-	246	hypothetical protein	Vibrio phage vB_VhaS-VHB1(100%)	QKE60734.1	2.00E-51
74	42250	44268	-	2019	hypothetical protein	Vibrio phage SIO-2(100%)	YP_004957563.1	0.00E+00
75	44252	45046	-	795	hypothetical protein	Vibrio phage vB_VhaS-VHB1(100%)	QKE60732.1	0.00E+00
76	45065	45724	-	660	hypothetical protein	Vibrio phage vB_VhaS-VHB1(100%)	QKE60731.1	6.00E-162
77	45798	46550	-	753	hypothetical protein	Vibrio phage SIO-2(100%)	YP_004957566.1	0.00E+00
78	46708	47562	-	855	hypothetical protein	Vibrio phage vB_VhaS-VHB1(99.65%)	QKE60729.1	0.00E+00
79	47831	48388	-	558	ribonuclease	Vibrio phage vB_VhaS-VHB1(99.46%)	QKE60728.1	2.00E-135
80	48388	49377	-	990	exonuclease	Vibrio phage SIO-2(100%)	YP_004957569.1	0.00E+00
81	49370	49822	-	453	Holliday junction resolvase	Vibrio phage SIO-2(100%)	YP_004957570.1	4.00E-107
82	50884	51297	-	414	hypothetical protein	Vibrio phage SIO-2(100%)	YP_004957571.1	2.00E-97

83	51304	51711	-	408	transcriptional regulator	Vibrio phage SIO-2(99.26%)	YP_004957572.1	1.00E-95
84	51789	53213	-	1425	hypothetical protein	Vibrio phage SIO-2(100%)	YP_004957573.1	0.00E+00
85	53261	54199	-	939	hypothetical protein	Vibrio phage vB_VhaS-VHB1(100%)	QKE60721.1	0.00E+00
86	54214	55197	-	984	hypothetical protein	Vibrio phage SIO-2(100%)	YP_004957575.1	0.00E+00
87	55218	55838	-	621	hypothetical protein	Vibrio phage vB_VhaS-VHB1(98.85%)	QKE60719.1	3.00E-139
88	55910	57412	-	1503	DNA helicase	Vibrio phage vB_VhaS-VHB1(99.80%)	QKE60718.1	0.00E+00
89	57412	57987	-	576	hypothetical protein	Vibrio phage SIO-2(100%)	YP_004957578.1	1.00E-140
90	57993	59468	-	1476	DNA binding protein	Vibrio phage vB_VhaS-VHB1(99.80%)	QKE60716.1	0.00E+00
91	59837	61075	-	1239	ATPase	Vibrio phage SIO-2(99.52%)	YP_004957580.1	0.00E+00
92	61203	61751	-	549	transmembrane helix protein	Vibrio phage vB_VhaS-VHB1(100%)	QKE60714.1	2.00E-128
93	61757	62185	-	429	hypothetical protein	Vibrio phage SIO-2(100%)	YP_004957582.1	1.00E-101
94	62178	62402	-	225	hypothetical protein	Vibrio phage vB_VhaS-VHB1(100%)	QKE60712.1	3.00E-43

95	62402	62701	-	300	hypothetical protein	Vibrio phage SIO-2(100%)	QKE60711.1	2.00E-65
96	62701	63207	-	507	hypothetical protein	Vibrio phage SIO-2(98.82%)	YP_004957585.1	7.00E-115
97	63211	64494	-	1284	hypothetical protein	Vibrio phage vB_VhaS-VHB1(99.30%)	QKE60709.1	0.00E+00
98	64500	65396	-	897	hypothetical protein	Vibrio phage vB_VhaS-VHB1(99.67%)	QKE60708.1	0.00E+00
99	65409	66380	-	972	hypothetical protein	Vibrio phage vB_VhaS-VHB1(99.69%)	QKE60707.1	0.00E+00
100	66393	66773	-	381	hypothetical protein	Vibrio phage SIO-2(100%)	YP_004957589.1	2.00E-85
101	66773	70903	-	4131	tail tape measure protein	Vibrio phage vB_VhaS-VHB1(100%)	QKE60705.1	0.00E+00
102	71107	71538	-	432	tail protein	Vibrio phage SIO-2(99.31%)	YP_004957591.1	5.00E-96
103	71619	72413	-	795	major tail protein	Vibrio phage vB_VhaS-VHB1(100%)	QKE60702.1	0.00E+00
104	72464	72919	-	456	hypothetical protein	Vibrio phage SIO-2(100%)	YP_004957593.1	4.00E-108
105	72919	73377	-	459	hypothetical protein	Vibrio phage SIO-2(100%)	YP_004957594.1	8.00E-107
106	73377	73997	-	621	hypothetical protein	Vibrio phage SIO-2(100%)	YP_004957595.1	1.00E-150

107	74010	74336	-	327	hypothetical protein	Vibrio phage vB_VhaS-VHB1(100%)	QKE60698.1	3.00E-73
108	74423	75364	-	942	major capsid protein	Vibrio phage vB_VhaS-VHB1(100%)	QKE60697.1	0.00E+00
109	75450	76622	-	1173	transport and binding protein	Vibrio phage vB_VhaS-VHB1(99.74%)	QKE60696.1	0.00E+00
110	76622	77233	-	612	protease	Vibrio phage SIO-2(100%)	YP_004957599.1	1.00E-146
111	77353	77685	-	333	hypothetical protein	Vibrio phage vB_VhaS-VHB1(100%)	QKE60694.1	4.00E-73
112	77756	79417	-	1662	hypothetical protein	Vibrio phage vB_VhaS-VHB1(100%)	QKE60693.1	0.00E+00
113	79413	80222	-	810	adenylosuccinate synthase	Vibrio phage vB_VhaS-VHB1(100%)	QKE60692.1	0.00E+00
114	80235	80504	-	270	hypothetical protein	Vibrio phage SIO-2(98.89%)	YP_004957603.1	3.00E-60

Supplement Table S6. General features of the putative ORFs identified in VPpYZU110

ORFs	Start	End	Strand	Length	Putative function	Best phage homolog(Identity)	Accession no.	E_value
1	7909	8262	+	354	lysozyme	Vibrio phage VAP7 (100.00%)	YP_009845677.1	3.00E-79
2	8966	11233	+	2268	ribonucleotide reductase of class Ia (aerobic), alpha subunit	Vibrio phage BX-1(100.00%)	UFD98110.1	0
3	11301	12392	+	1092	ribonucleoside diphosphate reductase small subunit	Vibrio phage VAP7 (100.00%)	YP_009845680.1	0
4	13962	14456	+	495	membrane lipoprotein	Vibrio phage vB_VpaS_SD25(100.00%)	XHB37237.1	3.00E-121
5	15898	16227	+	330	phosphatase	Vibrio phage VAP7(100.00%)	YP_009845687.1	1.00E-74
6	16632	17462	+	831	thymidylate synthase	Vibrio phage VAP7 (100.00%)	YP_009845688.1	0.00E+00
7	19621	19734	+	114	dihydrofolate reductase	Vibrio phage VAP7 (100.00%)	YP_009845692.1	5.00E-18
8	21923	22180	-	258	DUF4326 domain-containing protein	Vibrio phage VAP7 (98.84%)	YP_009845697.1	4.00E-57
9	33490	34503	-	1014	P-loop containing nucleoside triphosphate hydrolase	Vibrio phage VP-1 (99.41%)	AWY10106.1	0.00E+00

10	47290	48285	-	996	DNA primase	Vibrio phage VAP7 (99.70%)	YP_009845759.1	0.00E+00
11	54067	55932	-	1866	DNA topoisomerase II	Vibrio phage VAP7 (99.84%)	YP_009845769.1	0
12	59147	59800	-	654	RIIB lysis inhibitor	Vibrio phage VAP7 (100.00%)	YP_009845775.1	2.00E-159
13	62361	63431	-	1071	RIIA lysis inhibitor	Vibrio phage VAP7 (88.52%)	YP_009845776.1	0
14	64411	65697	-	1287	DNA primase/helicase	Vibrio phage VAP7 (100.00%)	YP_009845779.1	0.00E+00
15	66116	67132	-	1017	DNA repair protein	Vibrio phage VAP7 (100.00%)	YP_009845781.1	0.00E+00
16	69465	72404	-	2940	DNA polymerase	Vibrio phage vB_VpaS_SD25 (90.92%)	XHB37329.1	0
17	78868	79305	+	438	baseplate wedge subunit	Vibrio phage VAP7 (100.00%)	YP_009845793.1	2.00E-97
18	88593	89066	+	474	neck protein	Vibrio phage VAP7 (100.00%)	YP_009845801.1	1E-112
19	89252	89827	+	576	proximal tail sheath stabilization protein	Vibrio phage VP-1(99.48%)	AWY10142.1	8E-141
20	90577	92571	+	1995	terminase large subunit	Vibrio phage VAP7 (95.36%)	YP_009845804.1	0
21	94860	95231	+	372	tail tube protein	Vibrio phage VAP7 (100.00%)	YP_009845806.1	1.00E-85

22	97012	98397	+	1386	capsid assembly protein	Vibrio phage VAP7 (99.78%)	YP_009845808.1	0.00E+00
23	98974	99375	+	402	head maturation protease	Salmonella phage Vi01(71.64%)	YP_004327525.1	6.00E-65
24	99600	100259	+	660	prohead core protein	Vibrio phage VAP7 (100.00%)	YP_009845812.1	2.00E-156
25	100993	101838	+	846	major capsid protein	Vibrio phage VAP7 (100.00%)	YP_009845813.1	0.00E+00
26	104230	105750	+	1521	DNA helicase	Vibrio phage VAP7 (100.00%)	YP_009845818.1	0.00E+00
27	107096	107842	+	747	clamp loader of DNA polymerase	Vibrio phage VAP7 (100.00%)	YP_009845820.1	0.00E+00
28	108623	108952	+	330	translation repressor protein	Vibrio phage VAP7 (100.00%)	YP_009845822.1	2.00E-76
29	114092	114802	+	711	DNA endonuclease	Vibrio phage VAP7 (100.00%)	YP_009845830.1	2.00E-178
30	115368	117845	+	2478	coil containing protein	Vibrio phage VAP7 (77.59%)	YP_009845831.1	0.00E+00
31	118314	119648	+	1335	hypothetical protein	Vibrio phage BX-1 (99.77%)	UFD98149.1	0.00E+00
32	127758	128201	-	444	head completion protein	Vibrio phage VP-1(100.00%)	AWY10175.1	6.00E-107
33	129387	129548	+	162	baseplate wedge protein	Vibrio phage VAP7(100.00%)	YP_009845841.1	3.00E-30

34	134435	135157	-	723	putative DNA ligase	Vibrio phage VAP7 (100.00%)	YP_009845847.1	4.00E-175
35	140124	140528	-	405	single stranded DNA binding protein	Vibrio phage VP-1(100.00%)	AWY10185.1	4.00E-93
